# Supplementary material for: A generative model for inorganic materials design
Source: Nature. 2025 Jan 16;639(8055):624–32. doi: 10.1038/s41586-025-08628-5 (PMC11922738; doi:10.1038/s41586-025-08628-5)
Supplement: Supplementary file 1 — This Supplementary Information file contains the following sections: (A) Diffusion model for periodic materials, (B) Fine-tuning the score network for property-guided generation, (C) Dataset generation and (D) Results. [file 41586_2025_8628_MOESM1_ESM.pdf]

nature portfolio
https://doi.org/10.1038/s41586-025-08628-5

Supplementary information

A generative model for inorganic materials design

In the format provided by the authors and unedited

Supplementary Information

Table of Contents

A Diffusion model for periodic materials **2**
A.1 Representation of periodic materials 2
A.2 Invariance and equivariance in periodic materials 3
A.3 Diffusion model background and notation 3
A.4 Joint diffusion process 5
A.5 Atom type diffusion 5
A.6 Coordinate diffusion 8
A.7 Lattice diffusion 10
A.8 Architecture of the score network 12
A.9 Training loss 15
A.10 Ablation study 15
A.11 Comparison with CDVAE and DiffCSP 16

B Fine-tuning the score network for property-guided generation **18**
B.1 Fine-tuning the score network with adapter modules 18
B.2 Classifier-free guidance 19

C Dataset generation **20**
C.1 Data sources 20
C.2 DFT details 21

D Results **22**
D.1 Common experimental details 22
D.2 Qualitative analysis of generated structures 24
D.3 Stability, uniqueness and novelty (S.U.N.) 27
D.4 Ordered-disordered structure matcher 28
D.5 Generating stable and diverse materials 32
D.6 Generating materials with target chemistry 35
D.7 Designing materials with target symmetry 39
D.8 Designing materials with target magnetic, electronic and mechanical properties 41
D.9 Designing low-supply-chain risk magnets 44
D.10 Experimental validation of bulk modulus conditional generation . . 48

A Diffusion model for periodic materials

This section contains additional model details, following the notation listed in Table A1.

General notation	
$n \in \mathbb{N}$	Number of atoms in a crystal
$\boldsymbol{M} = (\boldsymbol{X}, \boldsymbol{A}, \boldsymbol{L})$	A crystal structure
$\boldsymbol{X} \in [0,1)^{3\times n}$	Fractional atomic coordinates
$\tilde{\boldsymbol{X}} \in \mathbb{R}^{3\times n}$	Cartesian atomic coordinates
$\boldsymbol{A} \in \mathbb{A}^n$	Atomic species in a crystal
$\boldsymbol{L} = (\boldsymbol{l}^1, \boldsymbol{l}^2, \boldsymbol{l}^3) \in \mathbb{R}^{3\times 3}$	The unit cell lattice matrix
$\boldsymbol{l}^j \in \mathbb{R}^3$, $j \in \{1,2,3\}$	The j-th lattice vector
$\boldsymbol{V} = (\boldsymbol{v}_1, \boldsymbol{v}_2, \dots \boldsymbol{v}_N) \in \mathbb{R}^{d\times N}$	Concatenation of N d-dimensional column vectors into a matrix
$\mathcal{E} \subset \{1,2,\dots,n\}^2 \times \mathbb{Z}^3$	Set of edges in a material
$i, j \in \{1,2,\dots,n\}$	Index of an atom in a material
$d \in \mathbb{N}$	The number of hidden dimension in our GNN
$\mathbf{1}_n \in \mathbb{R}^n$	n-dimensional column vector containing ones
**Diffusion notation**	
$t \in 1,2,\dots,T$	Diffusion timestep
$T \in \mathbb{N}$	Number of time discretization steps for the diffusion process
$q(\boldsymbol{x}_0)$	The data distribution
$q(\boldsymbol{x}_t \vert \boldsymbol{x}_{t-1})$	Single-step diffusion transition kernel
$q(\boldsymbol{x}_t \vert \boldsymbol{x}_0)$	One-shot diffusion kernel
$q(\boldsymbol{x}_T)$	Prior (noise) distribution
$\boldsymbol{s}_{\boldsymbol{\theta}}(\cdot, t)$	Score model
$\boldsymbol{s}_{\boldsymbol{X},\boldsymbol{\theta}}(\cdot, t)$	Score model for atomic coordinates
$\log p_{\boldsymbol{\theta}}(\boldsymbol{A}_0 \vert \boldsymbol{X}_t, \boldsymbol{L}_t, \boldsymbol{A}_t, t)$	Predicted logits for atom types at $t = 0$.
$\boldsymbol{s}_{\boldsymbol{L},\boldsymbol{\theta}}(\cdot, t)$	Score model for lattice
$\boldsymbol{z}$	Standard Gaussian noise $\boldsymbol{z} \sim \mathcal{N}(\boldsymbol{0}, \boldsymbol{I})$

Table A1: Table of notations

A.1 Representation of periodic materials

Any crystal structure can be represented by some repeating unit (called the *unit cell*) that tiles the entire 3D space. The unit cell itself contains a number of atoms that are arranged inside of it. Thus, we use the following universal representation for a material $\boldsymbol{M}$:

$$\boldsymbol{M} = (\boldsymbol{A}, \boldsymbol{X}, \boldsymbol{L}), \tag{A1}$$

where $\boldsymbol{A} = (a^1, a^2, \dots, a^n)^\top \in \mathbb{A}^n$ are the atomic species of the atoms inside the unit cell; $\boldsymbol{L} = (\boldsymbol{l}^1, \boldsymbol{l}^2, \boldsymbol{l}^3) \in \mathbb{R}^{3\times 3}$ is the lattice, i.e., the shape of the repeating unit cell; and $\boldsymbol{X} = (\boldsymbol{x}^1, \boldsymbol{x}^2, \dots, \boldsymbol{x}^n) \in [0,1)^{3\times n}$ are the *fractional* coordinates of the atoms inside the unit cell.

The lattice $\boldsymbol{L}$ is a parallelepiped defined by the three lattice vectors $\boldsymbol{l}^1, \boldsymbol{l}^2$, and $\boldsymbol{l}^3$. It can thus be compactly represented as a single 3×3 matrix with the three lattice vectors as its columns. The volume of a lattice is given by $\mathrm{Vol}(\boldsymbol{L}) = \lvert \det \boldsymbol{L} \rvert$. Any

physically sensible crystal must have a unit cell with nonzero volume, hence, we require any lattice matrix to be non-singular.

Fractional coordinates express the location of an atom using the lattice vectors as the basis vectors. For instance, an atom with fractional coordinates $\boldsymbol{x} = (0.2, 0.3, 0.5)^\top$ has Cartesian coordinates $\tilde{\boldsymbol{x}} = 0.2\boldsymbol{l}^1 + 0.3\boldsymbol{l}^2 + 0.5\boldsymbol{l}^3$. The periodicity in fractional coordinates is defined by the (flat) unit hypertorus, i.e., we have the equivalence relation $\boldsymbol{x} \sim \boldsymbol{x} + \boldsymbol{k}, \boldsymbol{k} \in \mathbb{Z}^3$. We can convert between fractional coordinates $\boldsymbol{X}$ and Cartesian coordinates $\tilde{\boldsymbol{X}}$ as follows:

$$\tilde{\boldsymbol{X}} = \boldsymbol{L}\boldsymbol{X}, \tag{A2}$$

$$\boldsymbol{X} = \boldsymbol{L}^{-1}\tilde{\boldsymbol{X}}. \tag{A3}$$

A.2 Invariance and equivariance in periodic materials

The energy per atom $\epsilon(\boldsymbol{M}) = E(\boldsymbol{M})/n$ of a periodic material $\boldsymbol{M} = (\boldsymbol{X}, \boldsymbol{L}, \boldsymbol{A})$ has several invariances.

- Permutation invariance: $\epsilon(\boldsymbol{X}, \boldsymbol{L}, \boldsymbol{A}) = \epsilon(\boldsymbol{P}(\boldsymbol{X}), \boldsymbol{L}, \boldsymbol{P}(\boldsymbol{A}))$ for every permutation matrix $\boldsymbol{P}$.
- Translation invariance: $\epsilon(\boldsymbol{X}, \boldsymbol{L}, \boldsymbol{A}) = \epsilon(\boldsymbol{X} + \boldsymbol{t}, \boldsymbol{L}, \boldsymbol{A})$ for every $\boldsymbol{t} \in \mathbb{R}^3$.
- Rotation invariance: $\epsilon(\boldsymbol{X}, \boldsymbol{L}, \boldsymbol{A}) = \epsilon(\boldsymbol{X}, \boldsymbol{R}(\boldsymbol{L}), \boldsymbol{A})$ for every rotation matrix $\boldsymbol{R} \in O(3)$.
- Periodic cell choice invariance: $\epsilon(\boldsymbol{X}, \boldsymbol{L}, \boldsymbol{A}) = \epsilon(\boldsymbol{C}^{-1}\boldsymbol{X}, \boldsymbol{L}\boldsymbol{C}, \boldsymbol{A})$, where $\boldsymbol{C}$ triangular with $\det \boldsymbol{C} = 1$ and $\boldsymbol{C} \in \mathbb{Z}^{3\times 3}$. See Fig. A2 for an example.
- Supercell invariance: $\epsilon(\boldsymbol{X}, \boldsymbol{L}, \boldsymbol{A}) = \epsilon\left(\bigoplus_{i=0}^{\det(\boldsymbol{C})} \boldsymbol{C}^{-1}(\boldsymbol{X} + \boldsymbol{k}_i\boldsymbol{1}_n^\top), \boldsymbol{L}\boldsymbol{C}, \bigoplus_{i=0}^{\det(\boldsymbol{C})} \boldsymbol{A}\right)$, where $\boldsymbol{C}$ is a 3×3 diagonal matrix with positive integers on the diagonal, $\boldsymbol{k}_i \in \mathbb{N}^3$ indexes the cell repetitions in the three lattice components, and $\bigoplus$ indicates concatenation.

Forces are instead equivariant to permutation and rotation, while being invariant to translation and periodic cell choice. Stress tensors are similarly invariant to permutation, translation, supercell choice, and periodic cell choice; while being equivariant to rotation (see Supplementary A.8.1 for additional details).

A.3 Diffusion model background and notation

Diffusion models [33, 34][1, 2] are a class of generative models that learn to revert a diffusion process. The diffusion process (also called the *forward* process) gradually corrupts an input sample $\boldsymbol{x}_0$ via transition kernels $q(\boldsymbol{x}_t|\boldsymbol{x}_{t-1})$[1], defining a Markov chain $\boldsymbol{x}_0 \to \boldsymbol{x}_1 \to \cdots \to \boldsymbol{x}_T$, where $T \in \mathbb{N}$ is the number of diffusion steps and $1 \leq t \leq T$. Here, we cover the typical case where the data is continuous-valued and the transition kernels are normal distributions. See Supplementary A.5 for details on discrete diffusion models.

[1] We follow the convention in machine learning literature that the functional forms of (conditional) probability density functions depend on the variables that appear as arguments. For example, $q(\boldsymbol{x}_t|\boldsymbol{x}_{t-1})$ could be written as $q_{\mathbf{X}_t|\mathbf{X}_{t-1}}(\boldsymbol{x}_t|\boldsymbol{x}_{t-1})$ to make the dependence of the functional form on t explicit, but we avoid this to prevent clutter.

The transition kernels are of the general form $q(\boldsymbol{x}_t|\boldsymbol{x}_{t-1}) = \mathcal{N}(f(\boldsymbol{x}_{t-1}, t), \sigma_t^2 \boldsymbol{I})$, where $f(\boldsymbol{x}_{t-1}, t) : \mathbb{R}^d \rightarrow \mathbb{R}^d$ is affine in $\boldsymbol{x}_{t-1}$. This implies that the one-shot transition kernel $q(\boldsymbol{x}_t|\boldsymbol{x}_0)$ is also Gaussian, and for popular choices $f(\cdot, t)$ the mean and variance are known in closed form. This enables us to efficiently obtain a noisy sample $\boldsymbol{x}_t$ at an arbitrary time step t during training.

Diffusion models are optimized to approximate the score function of the noise distributions $q(\boldsymbol{x}_t|\boldsymbol{x}_0)$ for any $1 \leq t \leq T$:

$$\boldsymbol{\theta}^* = \arg\min_{\boldsymbol{\theta}} \sum_{t=1}^{T} \sigma_t^2 \mathbb{E}_{q(\boldsymbol{x}_0)} \mathbb{E}_{q(\boldsymbol{x}_t|\boldsymbol{x}_0)} \left[\| \boldsymbol{s}_{\boldsymbol{\theta}}(\boldsymbol{x}_t, t) - \nabla_{\boldsymbol{x}_t} \log q(\boldsymbol{x}_t|\boldsymbol{x}_0) \|_2^2 \right], \tag{A4}$$

where $\boldsymbol{s}_{\boldsymbol{\theta}}(\boldsymbol{x}, t) : \mathbb{R}^d \times \mathbb{R}_+ \rightarrow \mathbb{R}^d$ is called the *score model*. It is standard practice [33, 34] to parameterize the model to predict the *noise* $\epsilon_t = -\sigma_t \nabla_{\boldsymbol{x}_t} \log q(\boldsymbol{x}_t|\boldsymbol{x}_0)$ instead of the score, since the magnitude of $\epsilon_t \sim \mathcal{N}(\boldsymbol{0}, \boldsymbol{I})$ is independent of the diffusion time step t.

The forward diffusion process is designed such that $q(\boldsymbol{x}_T|\boldsymbol{x}_0) \approx q(\boldsymbol{x}_T)$, where $q(\boldsymbol{x}_T)$ is a prior distribution that is easy to sample from (e.g., Gaussian).

In this work we leverage two popular diffusion processes for continuous data, i.e., the variance-exploding diffusion [33][3] and the variance-preserving diffusion [34][1] process, which we briefly explain in the following.

Variance-exploding diffusion

This is the diffusion process used in denoising score matching (DSM) [33]. We define a sequence of exponentially increasing standard deviations $\sigma_{\min} = \sigma_1, \ldots, \sigma_T = \sigma_{\max}$ that define the transition kernels:

$$q(\boldsymbol{x}_t|\boldsymbol{x}_{t-1}) = \mathcal{N}\left(\boldsymbol{x}_t, \left(\sigma_t^2 - \sigma_{t-1}^2\right) \boldsymbol{I}\right), \qquad q(\boldsymbol{x}_t|\boldsymbol{x}_0) = \mathcal{N}\left(\boldsymbol{x}_0, \sigma_t^2 \boldsymbol{I}\right). \tag{A5}$$

We can generate a sample using the learned model via annealed Langevin dynamics [33][3] or ancestral sampling from the graphical model $\prod_{t=1}^{T} p_{\boldsymbol{\theta}}\left(\boldsymbol{x}_{t-1}|\boldsymbol{x}_t\right)$ [35]:

$$\boldsymbol{x}_{t-1} = \boldsymbol{x}_t + (\sigma_t^2 - \sigma_{t-1}^2) \boldsymbol{s}_{\boldsymbol{\theta}^*}(\boldsymbol{x}_t, t) + \boldsymbol{z}\sqrt{\sigma_t^2 - \sigma_{t-1}^2}, \tag{A6}$$

where $\boldsymbol{x}_T \sim \mathcal{N}\left(\boldsymbol{0}, \sigma_T^2 \boldsymbol{I}\right)$, and $\boldsymbol{z} \sim \mathcal{N}\left(\boldsymbol{0}, \boldsymbol{I}\right)$ is standard Gaussian noise. In Supplementary A.6 we explain how we leverage variance-exploding diffusion in the diffusion process of the fractional coordinates.

Variance-preserving diffusion

This is the diffusion process used to train denoising diffusion probabilistic models (DDPMs) [34][1]. In variance-preserving diffusion we define a sequence of positive noise scales $0 < \beta_1, \beta_2, \ldots \beta_T < 1$ to obtain transition kernels of the form

$$q(\boldsymbol{x}_t|\boldsymbol{x}_{t-1}) = \mathcal{N}\left(\sqrt{1-\beta_t}\boldsymbol{x}_{t-1}, \beta_t \boldsymbol{I}\right), \qquad q(\boldsymbol{x}_t|\boldsymbol{x}_0) = \mathcal{N}\left(\sqrt{\bar{\alpha}_t}\boldsymbol{x}_0, (1-\bar{\alpha}_t)\boldsymbol{I}\right), \tag{A7}$$

where $\bar{\alpha}_t = \prod_{i=1}^{t}(1-\beta_t)$. Sampling from a model trained to revert the variance-preserving diffusion process also works via *ancestral sampling* from the graphical model $\prod_{t=1}^{T} p_{\boldsymbol{\theta}}(\boldsymbol{x}_{t-1}|\boldsymbol{x}_t)$:

$$\boldsymbol{x}_{t-1} = \frac{1}{\sqrt{1-\beta_t}}\left(\boldsymbol{x}_t + \beta_t \boldsymbol{s}_{\boldsymbol{\theta}^*}(\boldsymbol{x}_t, t)\right) + \sqrt{\beta_t}\boldsymbol{z}, \tag{A8}$$

starting from $\boldsymbol{x}_T \sim \mathcal{N}(\boldsymbol{0}, \boldsymbol{I})$, where $\boldsymbol{z} \sim \mathcal{N}(\boldsymbol{0}, \boldsymbol{I})$ is standard Gaussian noise. See Supplementary A.7 for details about how we leverage variance-preserving diffusion in the diffusion process of the lattice.

A.4 Joint diffusion process

To apply the construction of a diffusion process described in Supplementary A.3 to crystal structures described in Supplementary A.1, we define the forward process through a Markov chain $\boldsymbol{M}_0 \to \boldsymbol{M}_1 \to \cdots \to \boldsymbol{M}_T$ via a transition kernel that diffuses the atom coordinates, atom types, and the lattice independently as follows:

$$\begin{aligned} &q(\boldsymbol{A}_{t+1}, \boldsymbol{X}_{t+1}, \boldsymbol{L}_{t+1}|\boldsymbol{A}_t, \boldsymbol{X}_t, \boldsymbol{L}_t) \\ &\quad = q(\boldsymbol{A}_{t+1}|\boldsymbol{A}_t)q(\boldsymbol{X}_{t+1}|\boldsymbol{X}_t)q(\boldsymbol{L}_{t+1}|\boldsymbol{L}_t) \qquad (t = 0, 1, \ldots, T-1). \end{aligned} \tag{A9}$$

In addition, the noise distributions of atom species $\boldsymbol{A}$ and the fractional coordinates $\boldsymbol{X}$ factorize into the diffusion of the individual atoms:

$$q(\boldsymbol{A}_{t+1}|\boldsymbol{A}_t) = \prod_{i=1}^{n} q(a_{t+1}^i|a_t^i), \qquad q(\boldsymbol{X}_{t+1}|\boldsymbol{X}_t) = \prod_{i=1}^{n} q(\boldsymbol{x}_{t+1}^i|\boldsymbol{x}_t^i).$$

Note that the factorization of the forward diffusion process does not imply that the reverse diffusion process factorizes in the same way. Details of the atom type diffusion, coordinate diffusion, and lattice diffusion are described in Supplementary A.5 to A.7, respectively. The architecture of the score network $s_\theta(\boldsymbol{M}_t, t)$ is described in Supplementary A.8. The combined objective function is presented in Supplementary A.9.

A.5 Atom type diffusion

For the diffusion of the (discrete) atom species $\boldsymbol{A}$, we use the discrete denoising diffusion probabilistic model (D3PM) approach [2], which is a generalization of DDPMs to discrete data problems. As in DDPM, the forward diffusion process is a Markov process that gradually corrupts an input sample a_0, which is a scalar discrete random variable with K categories (e.g., atomic species):

$$q(a_{1:T}|a_0) = \prod_{t=1}^{T} q(a_t|a_{t-1}), \tag{A10}$$

where $a_0 \sim q(a_0)$ is an atomic species sampled from the data distribution and $a_T \sim q(a_T)$, where $q(a_T)$ is a prior distribution that is easy to sample from.

Denoting the one-hot representation of a as a row vector $\boldsymbol{a}$, we can express the transitions as:

$$q(\boldsymbol{a}_t|\boldsymbol{a}_{t-1}) = \mathrm{Cat}(\boldsymbol{a}_t; \boldsymbol{p} = \boldsymbol{a}_{t-1}\boldsymbol{Q}_t), \tag{A11}$$

where $[\boldsymbol{Q}_t]_{ij} = q(a_t = j|a_{t-1} = i)$ is the Markov transition matrix at time step t. $\mathrm{Cat}(\boldsymbol{a};\boldsymbol{p})$ is a categorical distribution over one-hot vectors whose probabilities are given by the row vector $\boldsymbol{p}$. Similar to DDPM, D3PM assumes that the forward diffusion factorizes over all discrete variables of a data point, i.e., all atomic species are diffused independently with the same transition matrices $\boldsymbol{Q}_t$. Hence, we only consider individual one-hot vectors in this section. D3PMs are trained by optimizing a variational lower bound:

$$\begin{aligned} L_{\mathrm{vb}} = \mathbb{E}_{q(\boldsymbol{a}_0)}\Bigg[& -\mathbb{E}_{q(\boldsymbol{a}_1|\boldsymbol{a}_0)} \log \tilde{p}_{\boldsymbol{\theta}}(\boldsymbol{a}_0|\boldsymbol{a}_1, 1) + D_{\mathrm{KL}}\left[q(\boldsymbol{a}_T|\boldsymbol{a}_0) \,||\, q(\boldsymbol{a}_T)\right] \\ & + \sum_{t=2}^{T} \mathbb{E}_{q(\boldsymbol{a}_t|\boldsymbol{a}_0)} D_{\mathrm{KL}}\left[q(\boldsymbol{a}_{t-1}|\boldsymbol{a}_t, \boldsymbol{a}_0) \,||\, p_{\boldsymbol{\theta}}(\boldsymbol{a}_{t-1}|\boldsymbol{a}_t, t)\right]\Bigg]. \end{aligned} \tag{A12}$$

Here, $\tilde{p}_{\boldsymbol{\theta}}(\boldsymbol{a}_0|\boldsymbol{a}_t, t)$ is the model's prediction of the "clean" atom types at $t = 0$, and $p_{\boldsymbol{\theta}}(\boldsymbol{a}_{t-1}|\boldsymbol{a}_t, t)$ is the single-step posterior (see Eq. (A16) and the surrounding paragraph for how $p_{\boldsymbol{\theta}}(\boldsymbol{a}_{t-1}|\boldsymbol{a}_t, t)$ is computed). Moreover, Austin et al. [2] propose an additional cross-entropy loss on the model's prediction $\tilde{p}_{\boldsymbol{\theta}}(\boldsymbol{a}_0|\boldsymbol{a}_t, t)$:

$$L_{\mathrm{CE}} = -\mathbb{E}_{q(\boldsymbol{a}_0)}\left[\sum_{t=2}^{T} \mathbb{E}_{q(\boldsymbol{a}_t|\boldsymbol{a}_0)} \log \tilde{p}_{\boldsymbol{\theta}}(\boldsymbol{a}_0|\boldsymbol{a}_t, t)\right],$$

so that the overall loss becomes

$$L = L_{\mathrm{vb}} + \lambda_{\mathrm{CE}} L_{\mathrm{CE}}. \tag{A13}$$

Three important characteristics of DDPM and DSM are that (1) given $\boldsymbol{x}_0$ we can sample noisy samples $\boldsymbol{x}_t$ for arbitrary t in constant time; (2) after sufficiently many diffusion steps, $\boldsymbol{x}_T$ follows a prior distribution that is easy to sample from; and (3) the posterior $q(\boldsymbol{x}_{t-1}|\boldsymbol{x}_t, \boldsymbol{x}_0)$ in Eq. (A12) is tractable and can be computed efficiently. D3PM also has these properties, as we briefly outline in the following:

(1) Fast sampling of $\boldsymbol{a}_t \sim q(\boldsymbol{a}_t|\boldsymbol{a}_0)$. Since the forward diffusion in D3PM is governed by discrete transition matrices $\{\boldsymbol{Q}_t\}_{t=1}^T$, we can write

$$q(\boldsymbol{a}_t|\boldsymbol{a}_0) = \mathrm{Cat}(\boldsymbol{a}_t; \boldsymbol{p} = \boldsymbol{a}_{t-1}\bar{\boldsymbol{Q}}_t), \qquad \text{where } \bar{\boldsymbol{Q}}_t = \boldsymbol{Q}_1\boldsymbol{Q}_2 \ldots \boldsymbol{Q}_t. \tag{A14}$$

The cumulative transition matrices $\bar{\boldsymbol{Q}}_t$ can be pre-computed and for many diffusion processes even have a closed form.

(2) Tractable prior distribution. Two of the proposed diffusion processes are the absorbing (which we employ in MatterGen) and uniform diffusion processes. Both gradually diffuse the data towards a limit distribution, which are the one-hot distribution on the absorbing state and the uniform distribution over all categories, respectively. For more details, we refer to Appendix A of Austin et al. [2].
(3) Tractable posterior $q(\boldsymbol{a}_{t-1}|\boldsymbol{a}_t, \boldsymbol{a}_0)$. Using Bayes' rule and exploiting the Markov property $q(\boldsymbol{a}_t|\boldsymbol{a}_{t-1}, \boldsymbol{a}_0) = q(\boldsymbol{a}_t|\boldsymbol{a}_{t-1})$, we can write

$$q(\boldsymbol{a}_{t-1}|\boldsymbol{a}_t, \boldsymbol{a}_0) = \frac{q(\boldsymbol{a}_t|\boldsymbol{a}_{t-1})q(\boldsymbol{a}_{t-1}|\boldsymbol{a}_0)}{q(\boldsymbol{a}_t|\boldsymbol{a}_0)}. \tag{A15}$$

All terms in Eq. (A15) can be computed efficiently in closed form given the forward diffusion process.

Reverse sampling process.

We generate a sample $\boldsymbol{a}_0$ by first sampling $\boldsymbol{a}_T$ and then gradually updating it to obtain $p_{\boldsymbol{\theta}}(\boldsymbol{a}_{0:T}) = q(\boldsymbol{a}_T) \prod_{t=1}^{T} p_{\boldsymbol{\theta}}(\boldsymbol{a}_{t-1}|\boldsymbol{a}_t, t)$. Austin et al. [2] propose to parameterize $p_{\boldsymbol{\theta}}(\boldsymbol{a}_{t-1}|\boldsymbol{a}_t, t)$ by predicting a distribution over $\boldsymbol{a}_0$ and then marginalizing it out:

$$p_{\boldsymbol{\theta}}(\boldsymbol{a}_{t-1}|\boldsymbol{a}_t, t) \propto \sum_{\boldsymbol{a}_0} q(\boldsymbol{a}_{t-1}, \boldsymbol{a}_t|\boldsymbol{a}_0)\tilde{p}_{\boldsymbol{\theta}}(\boldsymbol{a}_0|\boldsymbol{a}_t, t), \tag{A16}$$

where we can use our tractable posterior computation again. Since we have a discrete state space, marginalizing out $\boldsymbol{a}_0$ by explicit summation has complexity $\mathcal{O}(K)$. In the case of atomic species we have $K \simeq 100$; thus, this is relatively cheap. This parameterization has the advantage that potential sparsity in the diffusion process is efficiently enforced by using $q(\boldsymbol{a}_{t-1}, \boldsymbol{a}_t|\boldsymbol{a}_0)$ without having to be learned by the model.

Forward diffusion process.

As the specific flavor of D3PM forward diffusion we employ the masked diffusion process, which has shown best performance in the original study [2] as well as our initial experiments. Following Austin et al. [2], we introduce an extra atom species [MASK] at index $K-1$, which is the absorbing or masked state. At each timestep t, the transition matrices have the particularly simple form

$$[\boldsymbol{Q}_t^{\text{absorbing}}]_{ij} = \begin{cases} 1 & \text{if } i = j = m, \\ 1 - \beta_t & \text{if } i = j \neq m, \\ \beta_t & \text{if } j = m \neq i, \\ 0 & \text{if } m \neq i \neq j \neq m, \end{cases} \tag{A17}$$

where m corresponds to the absorbing state. Intuitively, each species has probability $1 - \beta_t$ of staying unchanged, and probability β_t of transitioning to the absorbing state. Once a species is absorbed, it can never leave that state, and there are no transitions between different non-masked atomic species. Thus, the limit distribution of this diffusion process is a point mass on the absorbing state.

A.6 Coordinate diffusion

For our model we perform diffusion on the *fractional* coordinates and outline the approach in the following. See Supplementary A.6.3 for a brief outline why we favor fractional coordinate diffusion over Cartesian. The fractional coordinates in a crystal structure live in a Riemannian manifold referred to as the flat torus $\mathbb{T}^3 = \mathbb{S}^1 \times \mathbb{S}^1 \times \mathbb{S}^1$, i.e., it is the quotient space $\mathbb{R}^3/\mathbb{Z}^3$ with equivalence relation:

$$\boldsymbol{x} + \boldsymbol{k} \sim \boldsymbol{x}, \qquad \boldsymbol{k} \in \mathbb{Z}^3. \tag{A18}$$

Thus, adding Gaussian noise to fractional coordinates naturally corresponds to sampling from a *wrapped* normal distribution, whose probability density is

$$\mathcal{N}_{\mathrm{W}}\left(\bar{\boldsymbol{x}}; \boldsymbol{x}, \sigma^2\boldsymbol{I}, \boldsymbol{I}\right) = \sum_{\boldsymbol{k}\in\mathbb{Z}^3} \mathcal{N}\left(\bar{\boldsymbol{x}}; \boldsymbol{x} - \boldsymbol{k}, \sigma^2\boldsymbol{I}\right), \tag{A19}$$

where $\mathcal{N}_{\mathrm{W}}(\boldsymbol{\mu}, \boldsymbol{\Sigma}, \boldsymbol{B})$ denotes a wrapped normal distribution with mean $\boldsymbol{\mu}$, covariance matrix $\boldsymbol{\Sigma}$, and periodic boundaries $\boldsymbol{B}$. If the periodic boundaries are $[0, 1)^3$, i.e., $\boldsymbol{B} = \boldsymbol{I}$, we write $\mathcal{N}_{\mathrm{W}}(\boldsymbol{\mu}, \boldsymbol{\Sigma})$ for brevity.

For the diffusion of the atom coordinates we use variance-exploding diffusion, i.e., the variance of the diffusion process increases exponentially with diffusion time. This has the advantage that the prior distribution $q(\boldsymbol{x}_T)$ is particularly simple, i.e., the uniform distribution in the range $[0, 1)^3$. Jing et al. [4] use this approach for torsional angles—which live in a 1D flat torus—in small molecule generation. The one-shot noising process of the fractional coordinates is therefore defined as

$$q(\boldsymbol{x}_t|\boldsymbol{x}_0) = \mathcal{N}_{\mathrm{W}}\left(\boldsymbol{x}_t; \boldsymbol{x}_0, \sigma_t^2\boldsymbol{I}\right). \tag{A20}$$

A.6.1 Variance adjustment for atomic density

One limitation of using a constant variance for the fractional coordinate diffusion is that the diffusion in Cartesian space will have difference variance depending on the size of the unit cell. This limitation becomes clear if we express the distribution of the Cartesian coordinates $\tilde{\boldsymbol{x}}_t$ using Eq. (A2) via linear transformation of a Gaussian random variable $\boldsymbol{x}_t$:

$$q(\tilde{\boldsymbol{x}}_t, |\boldsymbol{x}_0, \boldsymbol{L}_t) = \mathcal{N}_{\mathrm{W}}\left(\tilde{\boldsymbol{x}}_t; \boldsymbol{L}_t\boldsymbol{x}_0, \sigma_t^2\boldsymbol{L}_t\boldsymbol{L}_t^\top, \boldsymbol{L}_t\right). \tag{A21}$$

Observe that the covariance matrix $\boldsymbol{\Sigma}_t = \sigma_t^2\boldsymbol{L}_t\boldsymbol{L}_t^\top$ of the noisy Cartesian coordinates depends on the lattice. Thus, the (generalized) variance of the noise distribution also depends on the size of the unit cell, i.e., $|\det(\boldsymbol{\Sigma}_t)| = \left(\sigma_t^3|\det \boldsymbol{L}_t|\right)^2$.

We mitigate this effect by scaling the variance in fractional coordinate diffusion based on the size of the unit cell. Assuming roughly constant atomic density $\mathrm{d}(\boldsymbol{L}_t) = \frac{n}{\mathrm{Vol}(\boldsymbol{L}_t)} \propto 1 \Leftrightarrow \mathrm{Vol}(\boldsymbol{L}_t) = |\det \boldsymbol{L}_t| \propto n$. We therefore choose to scale σ_t accordingly, i.e.,

$$\sigma_t(n) = \frac{\sigma_t}{\sqrt[3]{n}}, \tag{A22}$$

such that $|\det(\mathbf{\Sigma}_t)| = \left(\frac{\sigma_t^3}{n}|\det \boldsymbol{L}_t|\right)^2$ is no longer proportional to n.

A.6.2 Score computation for fractional coordinates

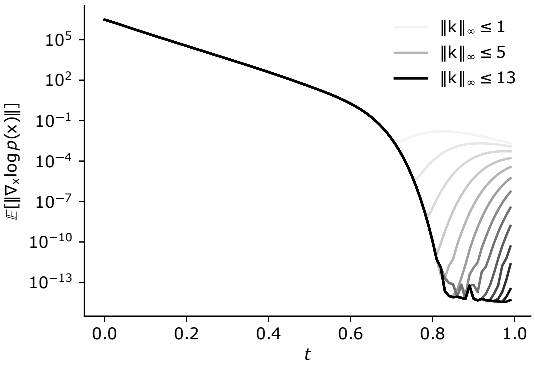

Fig. A1: Expected norm of the score of the wrapped normal distribution over diffusion time t for different truncation cutoffs $\|\boldsymbol{k}\|_\infty$, for a structure with $n = 10$ atoms. For $t > 0.7$, smaller truncation cutoffs incur larger approximation error, as their score norms do not approach zero for increasing t. We choose $\{\boldsymbol{k} \in \mathbb{Z}^3 \mid \|\boldsymbol{k}\|_\infty \leq 13\}$ as the set of $\boldsymbol{k}$ offset vectors in the truncated sum (Eq. (A23)) for our model training, which corresponds to $(2 * 13 + 1)^3 = 19,683$ terms in the truncated sum.

Recall from Eq. (A4) that training diffusion models requires computing the score function for the one-shot transition kernel. However, for the wrapped Normal distribution in Eq. (A19), (log-)likelihood and score computation are intractable because of the infinite sum. Given the thin tails of the normal distribution, both can be approximated reasonably well with a truncated sum (Fig. A1). More specifically, the score function of the isotropic wrapped Normal distribution can be expressed as

$$\nabla_{\bar{\boldsymbol{x}}} \log q_\sigma(\bar{\boldsymbol{x}}|\boldsymbol{x}) = -\sum_{\boldsymbol{k}\in\mathbb{Z}^3} w_{\boldsymbol{k}} \frac{\bar{\boldsymbol{x}} - \boldsymbol{x} + \boldsymbol{k}}{\sigma^2}, \tag{A23}$$

where

$$w_{\boldsymbol{k}} = \frac{1}{Z}\exp\left(-\frac{\|\bar{\boldsymbol{x}} - \boldsymbol{x} + \boldsymbol{k}\|^2}{2\sigma^2}\right), \qquad Z = \sum_{\boldsymbol{k}'\in\mathbb{Z}^3}\exp\left(-\frac{\|\bar{\boldsymbol{x}} - \boldsymbol{x} + \boldsymbol{k}'\|^2}{2\sigma^2}\right). \tag{A24}$$

A.6.3 Fractional vs Cartesian coordinate diffusion

Instead of diffusing fractional coordinates as in MatterGen, one could diffuse Cartesian coordinates, e.g., as done in CDVAE [4] (and in generative methods for molecules [5]).

However, this approach is not suitable for our framework. To see this, note that while in CDVAE the lattice $\boldsymbol{L}$ is fixed during the diffusion of the atom coordinates, we

diffuse the lattice simultaneously to the atom coordinates (and atomic species). This makes diffusion of Cartesian coordinates dependent on the lattice diffusion because the wrapped normal's covariance matrix and periodic boundaries at diffusion timestep t depend on knowing the lattice matrix $\boldsymbol{L}_t$. Consequently, our diffusion process from Eq. (A9) no longer factorizes into lattice and coordinates and needs to be adapted:

$$\begin{aligned} & q(\tilde{\boldsymbol{X}}_{t+1}, \boldsymbol{L}_{t+1}, \boldsymbol{A}_{t+1} | \tilde{\boldsymbol{X}}_t, \boldsymbol{L}_t, \boldsymbol{A}_t) \\ &= q(\tilde{\boldsymbol{X}}_{t+1} | \tilde{\boldsymbol{X}}_t, \boldsymbol{L}_{t+1}, \boldsymbol{L}_t) q(\boldsymbol{L}_{t+1} | \boldsymbol{L}_t) q(\boldsymbol{A}_{t+1} | \boldsymbol{A}_t). \end{aligned} \tag{A25}$$

Here, we need to condition $q(\tilde{\boldsymbol{X}}_{t+1})$ on $\boldsymbol{L}_{t+1}$ and $\boldsymbol{L}_t$ because in order to convert the Cartesian coordinates at time step t to time step $t+1$ we first need to convert $\tilde{\boldsymbol{x}}_t$ to fractional coordinates using $\boldsymbol{L}_t^{-1}$, and then to Cartesian coordinates at $t+1$ using $\boldsymbol{L}_{t+1}$.

The one-shot distribution of noisy Cartesian coordinates (similar to Eq. (A20) for the fractional case) becomes:

$$q(\tilde{\boldsymbol{x}}_t | \tilde{\boldsymbol{x}}_0, \{\boldsymbol{L}_{t'}\}_{t'=1}^{t}) = \mathcal{N}_{\mathrm{W}}\left(\tilde{\boldsymbol{x}}_t; \boldsymbol{L}_t \boldsymbol{L}_0^{-1} \tilde{\boldsymbol{x}}_0, \boldsymbol{L}_t \left(\sum_{t'=1}^{t} \sigma_{t'}^2 \boldsymbol{L}_{t'}^{-1} (\boldsymbol{L}_{t'}^{\top})^{-1}\right) \boldsymbol{L}_t^{\top}, \boldsymbol{L}_t\right). \tag{A26}$$

Observe that we require the entire trajectory of noisy lattices $\boldsymbol{L}_1, \ldots, \boldsymbol{L}_t$ in order to express the noise distribution of the Cartesian atomic coordinates. This means that we first need to sample the *entire* diffusion trajectory of the lattice, which is slow. Further, we have found computing the one-shot covariance matrix for the Cartesian coordinates to be numerically unstable for long diffusion trajectories. We therefore use the diffusion process of fractional coordinates described in the previous section.

A.7 Lattice diffusion

In addition to the diffusion of the atom types and coordinates described above, we also diffuse and denoise the lattice $\boldsymbol{L}$ in our approach. We use variance-preserving diffusion, as variance-exploding diffusion would lead to extremely large unit cells in the noisy limit. Those are challenging to handle for a graph neural network (GNN) with a fixed edge cutoff and would require the model to learn scores over a wide range of different length scales.

A.7.1 Fixed-rotation lattice diffusion

As the distribution of materials is invariant to global rotation, we can either choose a rotation-invariant prior distribution over unit cells, or decide on a canonical rotational alignment that we use throughout diffusion and denoising. We opt for the latter, as it gives us some more flexibility designing the diffusion process. Here, we choose to represent the lattice as a symmetric matrix. We can do so via the polar decomposition based on the singular value decomposition:

$$\tilde{\boldsymbol{L}} = \boldsymbol{U}\boldsymbol{L}, \qquad \boldsymbol{U} = \boldsymbol{W}\boldsymbol{V}^{\top}, \qquad \boldsymbol{L} = \boldsymbol{V}\boldsymbol{\Sigma}\boldsymbol{V}^{\top}, \tag{A27}$$

where $\boldsymbol{W}$ and $\boldsymbol{V}$ are the left and right singular vectors of $\tilde{\boldsymbol{L}}$, respectively, and $\boldsymbol{\Sigma}$ is the diagonal matrix of singular values. $\boldsymbol{U}$ is a rotation matrix and $\boldsymbol{L}$ is a symmetric positive-definite matrix.

We restrict our entire forward and reverse lattice diffusion to symmetric matrices, effectively eliminating the rotational degrees of freedom. That is, for an input data point with lattice matrix $\tilde{\boldsymbol{L}}$ with arbitrary rotation we first compute the symmetric lattice $\boldsymbol{L}$ via Eq. (A27) and only use $\boldsymbol{L}$ going forward. In the diffusion process, we corrupt lattices with additive Gaussian noise $\boldsymbol{z}$. However, naively sampling $\boldsymbol{z} \in \mathbb{R}^{3\times3} \sim \mathcal{N}(\mathbf{0}, \boldsymbol{I})$ leads to non-symmetric noise matrices in general, which means that after addition to $\boldsymbol{L}$ the noisy lattice matrices are not symmetric. To avoid this, we can sample $\hat{\boldsymbol{z}} \in \mathbb{R}^6 \sim \mathcal{N}(\mathbf{0}, \boldsymbol{I})$ and construct a symmetric noise matrix via

$$\boldsymbol{z} = \begin{bmatrix} \hat{\boldsymbol{z}}_1 & \hat{\boldsymbol{z}}_4 & \hat{\boldsymbol{z}}_5 \\ \hat{\boldsymbol{z}}_4 & \hat{\boldsymbol{z}}_2 & \hat{\boldsymbol{z}}_6 \\ \hat{\boldsymbol{z}}_5 & \hat{\boldsymbol{z}}_6 & \hat{\boldsymbol{z}}_3 \end{bmatrix}. \tag{A28}$$

In our entire forward and reverse lattice diffusion processes we exclusively use symmetric input lattice matrices $\boldsymbol{L}$ as well as symmetric noise matrices as described above, so that all intermediate noisy lattice matrices are symmetric as well.

A.7.2 Lattice diffusion with custom limit mean and variance

Naively using variance-preserving diffusion independently on the lattice vectors leads to the following forward diffusion distribution:

$$q(\boldsymbol{L}_t|\boldsymbol{L}_0) = \mathcal{N}\left(\sqrt{\bar{\alpha}_t}\boldsymbol{L}_0, (1-\bar{\alpha}_t)\boldsymbol{I}\right). \tag{A29}$$

However, for large t we observed that the majority of the resulting unit cells have very small volume and steep angles, which means that the atoms are extremely densely packed inside the noisy cells. We therefore modify the diffusion process as follows:

$$q(\boldsymbol{L}_t|\boldsymbol{L}_0) = \mathcal{N}\left(\sqrt{\bar{\alpha}_t}\boldsymbol{L}_0 + (1-\sqrt{\bar{\alpha}_t})\mu(n)\boldsymbol{I}, (1-\bar{\alpha}_t)\sigma_t^2(n)\boldsymbol{I}\right), \tag{A30}$$

which yields the following limit distribution for $T \to \infty$:

$$q(\boldsymbol{L}_T) = \mathcal{N}\left(\mu(n)\boldsymbol{I}, \sigma_T^2(n)\boldsymbol{I}\right). \tag{A31}$$

Thus, in the limit distribution we have a tendency towards cubic lattices $\boldsymbol{L} \propto \boldsymbol{I}$, which often occur in nature and have a relatively narrow range of volumes. Further, the lattice vector angles when sampling from the prior are mostly concentrated between 60° and 120°. This aligns both with the range of angles of Niggli-reduced cells as well as the initialization range of cell vector angles in RSS [43], suggesting that this is a good starting point for the generation process.

To understand the choice of the mean in the limit distribution, recall that the volume of a parallelepiped $\boldsymbol{L}$ is $|\det \boldsymbol{L}|$. We introduce a scalar coefficient $\mu(n)$ that depends on the number of atoms in the cell to make the atomic density of the mean

noisy lattice roughly constant for differently sized systems. Setting $\mu(n) = \sqrt[3]{nc}$, the volume of the prior mean becomes $\mathrm{Vol}(\sqrt[3]{nc}\boldsymbol{I}) = nc$. Thus, the atomic density of the prior mean becomes $\mathrm{d}(\mu(n)\boldsymbol{I}) = \frac{n}{\mathrm{Vol}(\mu(n)\boldsymbol{I})} = \frac{1}{c}$. We can set c to the inverse average density of the dataset as a reasonable prior.

Similarly, we adjust the variance in the limit distribution to be proportional to the volume, such that the signal-to-noise ratio of the noisy lattices is constant across the numbers of nodes. To this end, we set the limit standard deviation as $\sigma(n) = \sqrt[3]{n\nu}$. Thus, for a diagonal entry of the lattice matrix the signal-to-noise-ratio in the limit is $\lim_{t\to\infty} \frac{|\mu(n)|}{\sigma(n)} = \frac{\sqrt[3]{nc}}{\sqrt[3]{n\nu}} = \left(\frac{c}{\nu}\right)^{1/3}$ and therefore independent of the number of atoms.

A.8 Architecture of the score network

We employ an SE(3)-equivariant GNN to predict scores for the lattice, atom positions, and atom types in the denoising process. In particular, we adapt the GemNet architecture by Gasteiger et al. [6], originally developed to be a universal MLFF. GemNet is a symmetric message-passing GNN that uses directional information to achieve SO(3)-equivariance, and incorporates two- and three-body information in the first layer for efficiency. Since we do not predict energies, we adapt the direct (i.e., non-conservative) force prediction variant of the model, named GemNet-dT, which has been shown to be more computationally efficient and accurate in these scenarios [6]. We employ four message-passing layers, a cutoff radius of 7 Å for the neighbor list construction, and set the dimension of hidden representations for nodes and edges to 512.

We train the model to predict Cartesian coordinate scores $\boldsymbol{s}_{\boldsymbol{X},\boldsymbol{\theta}}(\boldsymbol{X}_t, \boldsymbol{L}_t, \boldsymbol{A}_t, t)$ as if they were non-conservative forces. These scores are equivariant to rotation and permutation, and invariant to translation. We then transform the Cartesian scores into fractional scores following Eq. (A2).

For the atom-type reverse diffusion, recall from Eq. (A16) that we predict the atom types $\boldsymbol{A}_0$ given the atom types $\boldsymbol{A}_t$ at time t. For materials, we additionally condition on lattice $\boldsymbol{L}_t$ and coordinates $\boldsymbol{X}_t$; more precisely, the (unnormalized) log-probabilities $\log p_{\boldsymbol{\theta}}(\boldsymbol{A}_0|\boldsymbol{X}_t, \boldsymbol{L}_t, \boldsymbol{A}_t, t)$ of the atomic species at $t = 0$ are computed as:

$$\log p_{\boldsymbol{\theta}}(\boldsymbol{A}_0|\boldsymbol{X}_t, \boldsymbol{L}_t, \boldsymbol{A}_t, t) = \boldsymbol{H}^{(L)}\boldsymbol{W}, \tag{A32}$$

where $\boldsymbol{H}^{(L)} \in \mathbb{R}^{n\times d}$ are the hidden representations of atoms at the last message-passing layer L, and $\boldsymbol{W} \in \mathbb{R}^{d\times K}$ are the weights of a fully-connected linear layer, with K being the number of atom types (including the masked null state).

A.8.1 Computation of the predicted lattice scores

To predict the lattice scores $\boldsymbol{s}_{\boldsymbol{L},\boldsymbol{\theta}}(\boldsymbol{X}_t, \boldsymbol{L}_t, \boldsymbol{A}_t, t)$, we utilize the model's hidden representations of the edges. For layer l, we denote the edge representation of the edge $(ij\boldsymbol{k}) \in \mathcal{E}$ between atoms i and j as $\boldsymbol{m}^l_{ij\boldsymbol{k}} \in \mathbb{R}^d$, where i is inside the unit cell and j is $\boldsymbol{k} \in \mathbb{Z}^3$ unit cells displaced from the center unit cell. We use a multi-layer perceptron (MLP) $\phi^l : \mathbb{R}^d \to \mathbb{R}$ to predict a scalar score per edge. We treat this as a prediction by the model indicating by how much an edge's length should increase or decrease, and

translate this into a predicted transformation of the lattice via chain rule derivation:

$$\begin{aligned}\frac{\partial \tilde{d}_{ij\boldsymbol{k}}}{\partial \boldsymbol{L}_t} &= \frac{\partial}{\partial \boldsymbol{L}_t} \left\| \boldsymbol{L}_t \left(\boldsymbol{x}_t^j - \boldsymbol{x}_t^i + \boldsymbol{k} \right) \right\|_2 \\ &= \frac{1}{\tilde{d}_{ij\boldsymbol{k}}} \boldsymbol{L}_t \left(\boldsymbol{x}_t^j - \boldsymbol{x}_t^i + \boldsymbol{k} \right) \cdot \left(\boldsymbol{x}_t^j - \boldsymbol{x}_t^i + \boldsymbol{k} \right)^\top \\ &= \frac{1}{\tilde{d}_{ij\boldsymbol{k}}} \tilde{\boldsymbol{d}}_{ij\boldsymbol{k}} (\boldsymbol{d}_{ij\boldsymbol{k}})^\top, \end{aligned} \tag{A33}$$

where $\tilde{d}_{ij\boldsymbol{k}} = \|\tilde{\boldsymbol{d}}_{ij\boldsymbol{k}}\|_2$ and $\tilde{\boldsymbol{d}}_{ij\boldsymbol{k}} = \boldsymbol{L}_t \left(\boldsymbol{x}_t^j - \boldsymbol{x}_t^i + \boldsymbol{k} \right)$ are the edge length and edge displacement in Cartesian coordinates, respectively, and $\boldsymbol{d}_{ij\boldsymbol{k}} = \boldsymbol{x}_t^j - \boldsymbol{x}_t^i + \boldsymbol{k}$ is the edge displacement in fractional coordinates. The predicted lattice score *per edge* is then $\phi^l(\boldsymbol{m}_{ij\boldsymbol{k}}^l) \cdot \frac{\partial \tilde{d}_{ij\boldsymbol{k}}}{\partial \boldsymbol{L}_t}$. These predicted scores are averaged over all edges $(ij\boldsymbol{k}) \in \mathcal{E}$ to get the predicted lattice score for layer l:

$$\hat{\boldsymbol{s}}_{\boldsymbol{L},\boldsymbol{\theta}}^l(\boldsymbol{X}_t, \boldsymbol{L}_t, \boldsymbol{A}_t, t) = \frac{1}{|\mathcal{E}|} \sum_{(ij\boldsymbol{k}) \in \mathcal{E}} \phi^l(\boldsymbol{m}_{ij\boldsymbol{k}}^l) \cdot \frac{1}{\tilde{d}_{ij\boldsymbol{k}}} \tilde{\boldsymbol{d}}_{ij\boldsymbol{k}} (\boldsymbol{d}_{ij\boldsymbol{k}})^\top. \tag{A34}$$

Stacking the model's predictions into a diagonal matrix $\boldsymbol{\Phi}^l \in \mathbb{R}^{|\mathcal{E}| \times |\mathcal{E}|} = \operatorname{diag}\left(\frac{\phi^l(\boldsymbol{m}_{ij\boldsymbol{k}}^l)}{|\mathcal{E}| \cdot \tilde{d}_{ij\boldsymbol{k}}} \right)$, we can write more concisely

$$\hat{\boldsymbol{s}}_{\boldsymbol{L},\boldsymbol{\theta}}^l(\boldsymbol{X}_t, \boldsymbol{L}_t, \boldsymbol{A}_t, t) = \tilde{\boldsymbol{D}} \boldsymbol{\Phi}^l \boldsymbol{D}^\top = \boldsymbol{L}_t \boldsymbol{D} \boldsymbol{\Phi}^l \boldsymbol{D}^\top, \tag{A35}$$

where $\tilde{\boldsymbol{D}}, \boldsymbol{D} \in \mathbb{R}^{3 \times |\mathcal{E}|}$ are the stacked matrices of Cartesian and fractional distance vectors, respectively, with $\tilde{\boldsymbol{D}} = \boldsymbol{L}_i \boldsymbol{D}$ for structure i. This form reveals that these predicted lattice scores have a key shortcoming. To see this, recall from Supplementary A.7.1 that we perform lattice diffusion on the subspace of *symmetric* lattice matrices. However, the lattice scores from $\hat{\boldsymbol{s}}_{\boldsymbol{L},\boldsymbol{\theta}}^l(\boldsymbol{X}_t, \boldsymbol{L}_t, \boldsymbol{A}_t, t)$ are generally not symmetric matrices. We address this issue with the following modification:

$$\begin{aligned}\boldsymbol{s}_{\boldsymbol{L},\boldsymbol{\theta}}^l(\boldsymbol{X}_t, \boldsymbol{L}_t, \boldsymbol{A}_t, t) &= \tilde{\boldsymbol{s}}_{\boldsymbol{L},\boldsymbol{\theta}}^l(\boldsymbol{X}_t, \boldsymbol{L}_t, \boldsymbol{A}_t, t) \boldsymbol{L}_t^\top \\ &= \boldsymbol{L}_t \boldsymbol{D} \tilde{\boldsymbol{\Phi}}^l \boldsymbol{D}^\top \boldsymbol{L}_t^\top = \tilde{\boldsymbol{D}} \tilde{\boldsymbol{\Phi}}^l \tilde{\boldsymbol{D}}^\top, \end{aligned} \tag{A36}$$

where $\tilde{\boldsymbol{\Phi}}^l = \operatorname{diag}\left(\frac{\phi^l(\boldsymbol{m}_{ij\boldsymbol{k}}^l)}{|\mathcal{E}| \cdot d_{ij\boldsymbol{k}}^2} \right)$. Finally, the predicted layer-wise lattice scores are summed to obtain the predicted lattice score:

$$\boldsymbol{s}_{\boldsymbol{L},\boldsymbol{\theta}}(\boldsymbol{X}_t, \boldsymbol{L}_t, \boldsymbol{A}_t, t) = \sum_{l=1}^{L} \boldsymbol{s}_{\boldsymbol{L},\boldsymbol{\theta}}^l(\boldsymbol{X}_t, \boldsymbol{L}_t, \boldsymbol{A}_t, t), \tag{A37}$$

which is scale-invariant and equivariant under rotation. The equivariance derives from the way it is composed with the Cartesian coordinate matrix, and the scale invariance

is due to the normalization happening inside $\tilde{\mathbf{\Phi}}^l$. In particular, the diagonal entries of $\tilde{\mathbf{\Phi}}^l$ related to the edges are normalized three times: they are divided by the total number of edges, and then multiplied twice by the inverse of the norm of the edge vectors. Given these properties, $\hat{\boldsymbol{s}}_{\boldsymbol{\theta}}$ behaves like a symmetric stress tensor $\boldsymbol{\sigma}$, since the stress tensor is scale-invariant and equivariant under the rotation operator $\boldsymbol{R}$:

$$\boldsymbol{\sigma}'(\lambda \boldsymbol{M}) = \boldsymbol{\sigma}(\boldsymbol{M}), \tag{A38}$$

$$\boldsymbol{\sigma}'(\boldsymbol{R}\boldsymbol{M}) = \boldsymbol{R}\boldsymbol{\sigma}(\boldsymbol{R}\boldsymbol{M})\boldsymbol{R}^\top, \tag{A39}$$

where we use λ to indicate the supercell replication operation.

A.8.2 Augmenting the input with lattice information

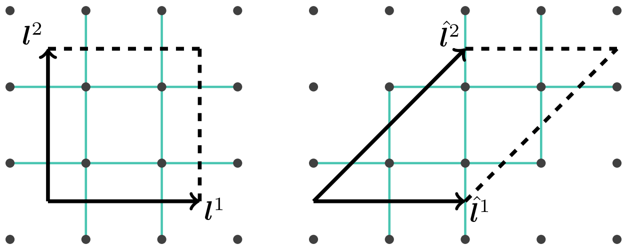

Fig. A2: Diagram showing two equivalent lattice choices $\boldsymbol{L}$ and $\hat{\boldsymbol{L}} = \boldsymbol{L}\boldsymbol{C}$ that lead to the same periodic structure. The dots represent atoms in the 2D periodic structures, and the blue lines indicate edges of atoms inside the unit cell to their four nearest neighbors, inside and outside the unit cell. Note that both choices of unit cell lead to indistinguishable structures, as indicated by the identical placement of atoms and equivalent edges. Here, $\boldsymbol{C} = \begin{bmatrix} 1 & 1 \\ 0 & 1 \end{bmatrix}$.

The chain-rule-based lattice score predictions from Eq. (A37) have shown to lack expressiveness for modeling the score of our Gaussian forward diffusion in our early experiments. We hypothesize that this is because our periodic GNN model is invariant to the particular choice of unit cell. For instance, it cannot distinguish the two structures in Fig. A2. To address this, we drop the invariance of the GNN w.r.t. equivalent choices of the unit cell by injecting information about the lattice angles into the internal representations. This means that the generative distribution is no longer invariant to the concrete choice of unit cell. We nonetheless note that any lattice can be *uniquely* transformed into its so-called Niggli-reduced cell [7]. We apply this transformation to all training data points and, consequently, side-step the loss of cell choice equivariance we introduce. Concretely, we concatenate the roto-translation invariant input edge representations $\boldsymbol{m}^{\text{inp}}$ with the cosines of the angles of the edge vectors

w.r.t. the lattice cell vectors:

$$\hat{\boldsymbol{m}}_{ij\boldsymbol{k}}^{\text{inp}} = \left(\boldsymbol{m}_{ij\boldsymbol{k}}^{\text{inp}},\ \cos(\boldsymbol{d}_{ij\boldsymbol{k}}, \boldsymbol{l}^1),\ \cos(\boldsymbol{d}_{ij\boldsymbol{k}}, \boldsymbol{l}^2),\ \cos(\boldsymbol{d}_{ij\boldsymbol{k}}, \boldsymbol{l}^3)\right). \tag{A40}$$

This additional information allows the model to distinguish the two cases in Fig. A2, while the internal representations remain invariant to rotation and translation.

A.9 Training loss

Our model is trained to minimize the following loss, which is a sum of the score matching loss (see Eq. (A4)) for the coordinates and cell, respectively, and the atom type loss (compare with D3PM objective in Eq. (A13)):

$$L = \lambda_{\text{coord}} L_{\text{coord}} + \lambda_{\text{cell}} L_{\text{cell}} + \lambda_{\text{types}} L_{\text{types}}, \tag{A41}$$

where

$$L_{\text{coord}} = \sum_{t=1}^{T} \sigma_t(n)^2 \mathbb{E}_{q(\boldsymbol{x}_0)} \mathbb{E}_{q(\boldsymbol{x}_t|\boldsymbol{x}_0)} \left[\|\boldsymbol{s}_{\boldsymbol{x},\boldsymbol{\theta}}(\boldsymbol{X}_t, \boldsymbol{L}_t, \boldsymbol{A}_t, t) - \nabla_{\boldsymbol{x}_t} \log q(\boldsymbol{x}_t|\boldsymbol{x}_0)\|_2^2 \right], \tag{A42}$$

$$L_{\text{cell}} = \sum_{t=1}^{T} (1 - \bar{\alpha}_t)\sigma_t(n)^2 \mathbb{E}_{q(\boldsymbol{L}_0)} \mathbb{E}_{q(\boldsymbol{L}_t|\boldsymbol{L}_0)} \left[\|\boldsymbol{s}_{\boldsymbol{L},\boldsymbol{\theta}}(\boldsymbol{X}_t, \boldsymbol{L}_t, \boldsymbol{A}_t, t) - \nabla_{\boldsymbol{L}_t} \log q(\boldsymbol{L}_t|\boldsymbol{L}_0)\|_2^2 \right] \tag{A43}$$

$$\begin{aligned} L_{\text{types}} = \mathbb{E}_{q(\boldsymbol{a}_0)} \Bigg[&\sum_{t=2}^{T} \mathbb{E}_{q(\boldsymbol{a}_t|\boldsymbol{a}_0)} [D_{\text{KL}} \left[q(\boldsymbol{a}_{t-1}|\boldsymbol{a}_t, \boldsymbol{a}_0) \,||\, p_{\boldsymbol{\theta}}(\boldsymbol{a}_{t-1}|\boldsymbol{X}_t, \boldsymbol{L}_t, \boldsymbol{A}_t)\right] \\ &- \lambda_{\text{CE}} \log p_{\boldsymbol{\theta}}(\boldsymbol{a}_0|\boldsymbol{X}_t, \boldsymbol{L}_t, \boldsymbol{A}_t, t)] - \mathbb{E}_{q(\boldsymbol{a}_1|\boldsymbol{a}_0)} \left[\log p_{\boldsymbol{\theta}}(\boldsymbol{a}_0|\boldsymbol{X}_1, \boldsymbol{L}_1, \boldsymbol{A}_1, 1)\right] \Bigg]. \end{aligned} \tag{A44}$$

For simplicity, Eqs. (A42) and (A44) show the loss only for a single atom's coordinates and specie, respectively; the overall losses for coordinates and atom types sum over all atoms in a structure.

A.10 Ablation study

In Table A2 we present an ablation study, demonstrating that the model innovations introduced in the previous sections improve results as measured by average RMSD and percentage of S.U.N. structures. With one exception, MatterGen-MP outperforms all ablated models on both metrics; the only exception is removing the scaling of the position noise σ based on the number of atoms from Supplementary A.6.1. Here, RMSD

Removed MatterGen feature	Replaced with	Avg. RMSD [Å]	% S.U.N.
MatterGen-MP		0.11	22.56
Variance adjustment (A.6.1)	Default wrapped VESDE	0.09	20.59
Symmetric lattice diffusion (A.7.1)	-	0.18	17.70
Wrapped normal score (A.6)	CDVAE heuristic score	1.41	5.18
Lattice angle augmentation (A.8.2)	-	1.32	2.16
D3PM (A.5)	VPSDE on one-hot types	0.58	4.32
Mean variance adjustment (A.7)	Standard VPSDE	-	-

Table A2: Ablation study results for MatterGen-MP model trained on MP-20.

of MatterGen-MP is slightly worse, but the fraction of S.U.N. structures is better. Since we argue that the fraction of S.U.N. structures corresponds more closely to real-world utility, we choose to add the modification from Supplementary A.6.1 into our final MatterGen architecture. Moreover, we show that replacing CDVAE's heuristic coordinate score with the wrapped Normal score increases the S.U.N. percentage by more than 4x and reduces RMSD by more than 10x. We further show that our custom lattice variance-preserving stochastic differential equation (VPSDE) is crucial; replacing it with a standard VPSDE (with limit distribution $\mathcal{N}(0, 1)$ for all lattice matrix elements), as used by Jiao et al. [12], leads to the sampling to fail because of exploding lattices. We attribute this to the fact that MatterGen's GNN backbone operates on Cartesian coordinates. Using a standard VPSDE for the lattice leads to extremely high-density prior samples at the beginning of samples, which a model using Cartesian coordinates struggles with. However, compared to DiffCSP—which uses fractional coordinates to avoid this problem—MatterGen's reliance on Cartesian coordinates leads to drastically improved RMSD results (Fig. 2 and Supplementary D.5.2). We attribute this to the interatomic distances used by our model being physically interpretable across all samples, whereas distances in fractional coordinates are only interpretable in combination with the lattice vectors.

A.11 Comparison with CDVAE and DiffCSP

The methods most closely related to MatterGen are CDVAE [4] and DiffCSP [12]. In this section, we explain the key differences between MatterGen and these methods.

One key difference between CDVAE and MatterGen is that the former is based on the variational autoencoder [8], while MatterGen is a diffusion model. Thus, CDVAE has an encoder and a decoder, where the decoder starts from a latent variable $\boldsymbol{z}$ and outputs a crystal structure $(\boldsymbol{A}, \boldsymbol{X}, \boldsymbol{L})$. The decoder consists of two submodules: one predicting aggregate properties (lattice parameters, number of atoms, relative proportions of elements), and a diffusion-based GNN whose task is to denoise the coordinates and atom types. We attribute MatterGen's stronger performance over CDVAE mostly to these reasons: (i) while the coordinate diffusion uses the wrapped Normal distribution (on Cartesian coordinates), CDVAE uses a heuristic target score, which essentially approximates Eq. (A23) with the single summand with highest $w_{\boldsymbol{k}}$, whereas MatterGen uses a close approximation via truncated sum; (ii) CDVAE predicts the lattice from the

latent variable $\boldsymbol{z}$ and never updates it during the denoising, which means that potential errors in the initial prediction cannot be corrected, whereas MatterGen denoises the lattice jointly with the coordinates and atom types; (iii) the prior regularization in CDVAE is weak, which leads to a divergence of predicted latent variables $\boldsymbol{z}$ of the training samples and the standard Normal prior; this mismatch leads to decreased performance when sampling from the standard Normal prior, e.g., the decoder predicts too many different elements on average compared to the training distribution (see number of elements results in Table 2 of [4]). In MatterGen, on the other hand, the forward diffusion process is defined such that it converges to the prior distribution, which is easy to sample from. In addition, as a diffusion model, MatterGen can leverage classifier-free guidance [37], the state-of-the-art conditional generation technique used in Dall·E 2 [48] and Stable Diffusion [9], whereas CDVAE has to resort to latent-space optimization.

DiffCSP, on the other hand, is also a pure diffusion model. Key differences to MatterGen are (i) the GNN backbone—MatterGen uses GemNet [6], whereas DiffCSP uses a simpler backbone similar to EGNN [10]; (ii) DiffCSP operates purely on fractional coordinates, whereas the MatterGen backbone uses Cartesian coordinates. We argue that Cartesian coordinates have important advantages over fractional coordinates, e.g., Cartesian interatomic distances remain unchanged when constructing a supercell, whereas fractional distances change even though the supercell is physically identical to the original structure. (iii) DiffCSP's main task is (unconditional) crystal structure prediction (CSP), i.e., predicting atom coordinates and the lattice given fixed element per atom. DiffCSP can also generate the atom types via diffusion, yet uses continuous diffusion of one-hot-encoded elements, whereas MatterGen uses D3PM [2], which was specifically designed for discrete data. MatterGen is also the first generative model for materials to leverage classifier-free guidance [37] for property-guided generation, which arguably is the key capability for real-world usefulness.

B Fine-tuning the score network for property-guided generation

Here we discuss our fine-tuning procedure of the score model to enable *property-guided* generation via classifier-free guidance [37].

B.1 Fine-tuning the score network with adapter modules

Leveraging the large-scale unlabeled Alex-MP-20 dataset enables MatterGen to generate a broad distribution of stable material structures via reverse diffusion, driven by unconditional scores. To facilitate conditional generation with classifier-free guidance, the property-conditioned scores need to be learned through a labeled dataset. However, labeled datasets, often limited in size and diversity, present challenges in learning the conditional scores from scratch.

To enable rapid learning of the conditional scores in the sparsely-labeled data regime, we propose to fine-tune the unconditional score network with additional trainable adapter modules. Each adapter layer is a combination of an MLP layer and a zero-initialized mix-in layer [36], so MatterGen still outputs the learned unconditional scores at initialization. This is desired because the unconditional scores have been optimized to generate stable materials during pre-training, which is a prerequisite for modeling the property-conditional distribution of materials.

The additional adapter modules consist of an embedding layer f_{embed} for the property label that outputs a property embedding $\boldsymbol{g}$, and a series of adapter layers, one before each message-passing layer (four in total). The adapter layer augments the atom embedding of the original GemNet score network to incorporate property information. Concretely, at the L-th interaction layer, given the property embedding $\boldsymbol{g}$ and the intermediate node hidden representation $\{\boldsymbol{H}_j^{(L)}\}_{j=1}^{n}$, the property-augmented node hidden representation $\{\boldsymbol{H}_j'^{(L)}\}_{j=1}^{n}$ is given by:

$$\boldsymbol{H}_j'^{(L)} = \boldsymbol{H}_j^{(L)} + f_{\text{mixin}}^{(L)}\left(f_{\text{adapter}}^{(L)}(\boldsymbol{g})\right) \cdot \mathbb{I}(\text{property is not null}), \tag{B45}$$

where $f_{\text{mixin}}^{(L)}$ is the L-th mix-in layer, which is a zero-initialized linear layer without bias weights. $f_{\text{adapter}}^{(L)}$ is the L-th adapter layer, which is a two-layer MLP model. The indicator function $\mathbb{I}(\text{property is not null})$ ensures the model outputs the unconditional score when no conditional label is given. The adapter modules add additional weights of f_{embed}, $f_{\text{adapter}}^{(L)}$, and $f_{\text{mixin}}^{(L)}$ to each layer of the model. Note that we update *all* neural network weights during fine-tuning, not just the added adapter and mix-in layers.

The fine-tuning process uses the same training objective as the unconditional pre-training stage with conditional property labels incorporated. When fine-tuning finishes, the score network is able to predict both conditional and unconditional scores. The fine-tuned model enables us to generate structures satisfying the property condition without a major sacrifice in terms of stability and novelty. With the unconditional score network as a strong initialization, the fine-tuning procedure is more computation- and sample-efficient than re-training from scratch if the labeled dataset is only sparsely labeled.

Finally, note that the above fine-tuning scheme also works for fine-tuning on multiple properties. A limitation of this approach is that we have to fine-tune a new model for every combination of properties. If we assume each property from some given set of properties is conditionally independent, we could instead compose multiple single-property conditional models together by following ideas from Liu et al. [11]. We leave this extension for future work.

B.2 Classifier-free guidance

To generate samples conditioned on a specific value c of a property, we adopt classifier-free diffusion guidance [37] throughout this work. In classifier-free guidance, a guidance factor γ is applied to the conditional distribution $p(\boldsymbol{M}_t|c)$, such that

$$\begin{aligned} p_\gamma(\boldsymbol{M}_t|c) &\propto p(c|\boldsymbol{M}_t)^\gamma p(\boldsymbol{M}_t) \\ &\propto \left(\frac{p(\boldsymbol{M}_t|c)}{p(\boldsymbol{M}_t)}\right)^\gamma p(\boldsymbol{M}_t) \\ &\propto p(\boldsymbol{M}_t|c)^\gamma p(\boldsymbol{M}_t)^{1-\gamma} \end{aligned} \tag{B46}$$

is used instead of $p(\boldsymbol{M}_t)$ when evaluating the model score during the reverse process in the conditional setting. We adopt a value of $\gamma = 2$ in all conditional generation experiments.

B.2.1 Continuous case

The conditional score follows from Eq. (B46) by taking gradients of the logarithm w.r.t. continuous variables in $\boldsymbol{M}_t$. For example, for fractional coordinates $\boldsymbol{X}_t$ we have

$$\nabla_{\boldsymbol{X}_t} \ln p_\gamma(\boldsymbol{X}_t|c) = \gamma \nabla_{\boldsymbol{X}_t} \ln q(\boldsymbol{X}_t|c) + (1-\gamma)\nabla_{\boldsymbol{X}_t} \ln q(\boldsymbol{X}_t). \tag{B47}$$

Practically, learning a conditional score $\nabla_{\boldsymbol{X}_t} \ln q(\boldsymbol{X}_t|c)$ equates to concatenating a latent embedding $\boldsymbol{g}_c \in \mathbb{R}^d$ of the condition c to the score model $\boldsymbol{s}_{\boldsymbol{\theta}}(\boldsymbol{M}_t, \boldsymbol{g}_c, t)$ during score matching. The unconditional score $\nabla_{\boldsymbol{X}_t} \ln p(\boldsymbol{X}_t)$ is obtained by providing a null embedding for the condition, i.e., using $\boldsymbol{s}_{\boldsymbol{\theta}}(\boldsymbol{M}_t, \boldsymbol{g}_c = \text{null}, t)$. When we condition on multiple properties, the conditional score for N properties with embeddings $\boldsymbol{g}_{c_i}$ is obtained by $\boldsymbol{s}_{\boldsymbol{\theta}}(\boldsymbol{M}_t, \boldsymbol{g}_{c_1}, \boldsymbol{g}_{c_2}, \ldots, \boldsymbol{g}_{c_N}, t)$.

B.2.2 Discrete case

The model's task in denoising discrete atom types $\boldsymbol{a}$ is to fit and predict $\tilde{q}(\boldsymbol{a}_{t-1}|\boldsymbol{a}_t, c)$. Following Eq. (4) in [2], we can rewrite this as

$$\tilde{q}(\boldsymbol{a}_{t-1}|\boldsymbol{a}_t, c) \propto \sum_{\boldsymbol{a}_0} q(\boldsymbol{a}_{t-1}, \boldsymbol{a}_t|\boldsymbol{a}_0) \cdot \tilde{q}(\boldsymbol{a}_0|\boldsymbol{a}_t, c).$$

Thus, the predictive task is to approximate $p_\theta(\boldsymbol{a}_0|\boldsymbol{a}_t, c) \approx \tilde{q}(\boldsymbol{a}_0|\boldsymbol{a}_t, c)$. For this distribution we can perform classifier-free guidance as follows:

$$\begin{aligned}\tilde{q}_\gamma(\boldsymbol{a}_0|\boldsymbol{a}_t, c) &\propto \tilde{q}(c|\boldsymbol{a}_0, \boldsymbol{a}_t)^\gamma \cdot \tilde{q}(\boldsymbol{a}_0|\boldsymbol{a}_t) \\ &= \left(\frac{\tilde{q}(\boldsymbol{a}_0|c, \boldsymbol{a}_t) \cdot \tilde{q}(c|\boldsymbol{a}_t)}{\tilde{q}(\boldsymbol{a}_0|\boldsymbol{a}_t)}\right)^\gamma \cdot \tilde{q}(\boldsymbol{a}_0|\boldsymbol{a}_t) \\ &\propto \tilde{q}(\boldsymbol{a}_0|c, \boldsymbol{a}_t)^\gamma \cdot \tilde{q}(\boldsymbol{a}_0|\boldsymbol{a}_t)^{1-\gamma}.\end{aligned}$$

We can approximate this guided distribution accordingly with an unconditional and a conditional prediction model, i.e., $p_\theta(\boldsymbol{a}_0|c, \boldsymbol{a}_t, t)^\gamma \cdot p_\theta(\boldsymbol{a}_0|\boldsymbol{a}_t, t)^{1-\gamma} \approx \tilde{q}(\boldsymbol{a}_0|c, \boldsymbol{a}_t)^\gamma \cdot \tilde{q}(\boldsymbol{a}_0|\boldsymbol{a}_t)^{1-\gamma}$. Taking the logarithm, we obtain

$$\log\left(p_\theta(\boldsymbol{a}_0|c, \boldsymbol{a}_t, t)^\gamma \cdot p_\theta(\boldsymbol{a}_0|\boldsymbol{a}_t, t)^{1-\gamma}\right) = \gamma \log p_\theta(\boldsymbol{a}_0|c, \boldsymbol{a}_t, t) + (1-\gamma)\log p_\theta(\boldsymbol{a}_0|\boldsymbol{a}_t, t).$$

C Dataset generation

Here we provide details about the training dataset Alex-MP-20 and the reference dataset Alex-MP-ICSD used throughout this work.

C.1 Data sources

We obtained crystal structures via three sources:

- MP (v2022.10.28, Creative Commons Attribution 4.0 International License) [14], an open-access resource containing DFT-relaxed crystal structures obtained from a variety of sources, but largely based upon experimentally-known crystals.
- The Alexandria dataset [25, 38] (Creative Commons Attribution 4.0 International License), an open-access resource containing DFT-relaxed crystal structures from a variety of sources, including a large quantity of hypothetical crystal structures generated by ML methods or other algorithmic means.
- ICSD (release 2023.1) [39], a proprietary database containing crystal structures refined from experiment. For the purposes of dataset generation, we queried ICSD only for experimental crystal structures that were not tagged as already included in MP, and that were directly calculable by DFT (i.e., ordered crystals).

For crystal structures from MP, we retrieved existing calculations via the MP API. For other data sources, we performed new calculations using MP settings to guarantee consistency of data (see Supplementary C.2). We then followed MP's data analysis approach as implemented in `emmet` [12], which includes the following steps:

1. Validation of each individual DFT calculation to ensure required minimum quality criteria are met.
2. Grouping of calculations of equivalent crystal structures, which de-duplicates crystal structures when the same crystal is present in multiple data sources. See Fig. C3 for an overview of the resulting statistics.
3. Application of an empirical correction scheme [13] to address known systematic errors from the Perdew–Burke–Ernzerhof (PBE) functional.

4. Construction of convex hull phase diagrams for each chemical system.

This process resulted in a reference dataset of 850,384 unique structures with associated energy above hull values calculated using DFT. We refer to this as the Alex-MP-ICSD dataset. This dataset was then used to derive the Alex-MP-20 dataset, whose element distribution is shown in Fig. C4. The Alex-MP-ICSD dataset is used as a reference for the computation of stability (i.e., energy above hull) of generated structures. We extend the Alex-MP-ICSD dataset to include 117,652 disordered structures coming from ICSD when computing novelty of generated structures, and we develop an *ordered-disordered* structure matcher to assess whether generated structures can be identified as ordered approximations of known disordered structures (Supplementary D.4). To train MatterGen, we employ a subset of the Alex-MP-ICSD dataset, selecting only structures with up to 20 atoms and whose energy above hull is below 0.1 eV/atom; we refer to this as the Alex-MP-20 dataset. Furthermore, we manually exclude from the Alex-MP-20 dataset all structures belonging to the "well-explored" chemical systems, as defined in Supplementary D.6. Additionally, we reserve structures present only in ICSD for testing purposes, and therefore exclude them from the Alex-MP-20 dataset. In Fig. C3, we report the structure provenance and quantity for the reference (Alex-MP-ICSD) and training (Alex-MP-20) datasets. Finally, the dataset employed to train the MatterGen-MP model contains structures from the MP-20 dataset (containing structures with up to 20 atoms) whose energy above hull is below 0.1 eV/atom from the reference convex hull.

C.2 DFT details

All DFT calculations were performed using the Vienna ab initio simulation package (VASP) [14] within the projector augmented-wave formalism via `atomate2`, `pymatgen` and `custodian` [15]. All parameters of the calculations were chosen to be consistent with MP [14], including use of the PBE functional [16] and Hubbard U corrections. Specifically, the following workflows were used for the calculation of associated properties:

- Structural relaxations and static calculations for total energy were calculated with the `MPRelaxSet` settings and via the `DoubleRelaxMaker` and `StaticMaker` classes.
- Band gaps were calculated as above, but via the `BandStructureMaker` class.
- Elastic tensors were calculated with the default `ElasticMaker` class with a stencil including -0.01 % and 0.01 % deformations, as specified by `wf_elastic_constant_minimal` in the `atomate` code. The "minimal" preset is used for reasons of computational efficiency.

All settings were previously benchmarked or in use by MP, and every effort was made to ensure consistent settings were used in the current work.

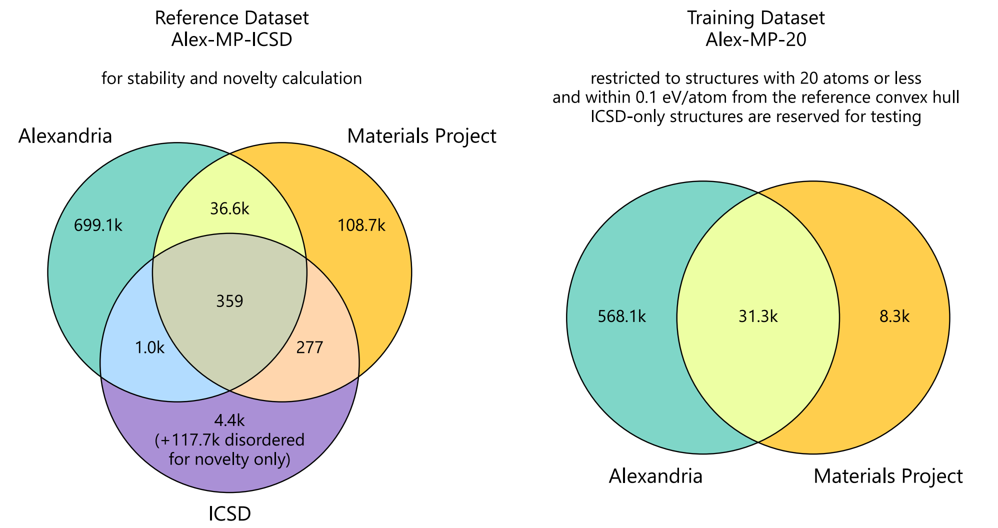

Fig. C3: Venn diagrams (not to scale) showing the overlap of crystal structures from different data sources for the Alex-MP-ICSD reference dataset (left) and the Alex-MP-20 training dataset (right). Crystal structures were de-duplicated after calculation and therefore the overlap in this diagram shows cases where the same crystal structure was present in multiple data sources. Note that the statistics for ICSD only include the structures sourced for this study and not the full ICSD database.

D Results

This section contains supplementary information for the results detailed in Section 2.

D.1 Common experimental details

In this section we provide information about settings used across different computational experiments, including training and sampling details of MatterGen, and additional information about the MLFF we employ. Details specific to certain computational experiments are deferred to Supplementary D.5 to D.10.

D.1.1 Hyperparameters for training base model

The base unconditional model was trained for 1.74 million steps with a batch size of 64 per GPU over eight A100 GPUs using the Adam optimizer [18]. The learning rate was initialized at 0.0001 and was decayed using the ReduceLROnPlateau scheduler with decay factor 0.6, patience 100 and minimum learning rate 10^{-6}. For the training loss we use $\lambda_{\text{coord}} = 0.1$ and $\lambda_{\text{cell}} = \lambda_{\text{types}} = 1$, as well as the recommended value $\lambda_{\text{CE}} = 0.01$ for the D3PM cross-entropy loss.

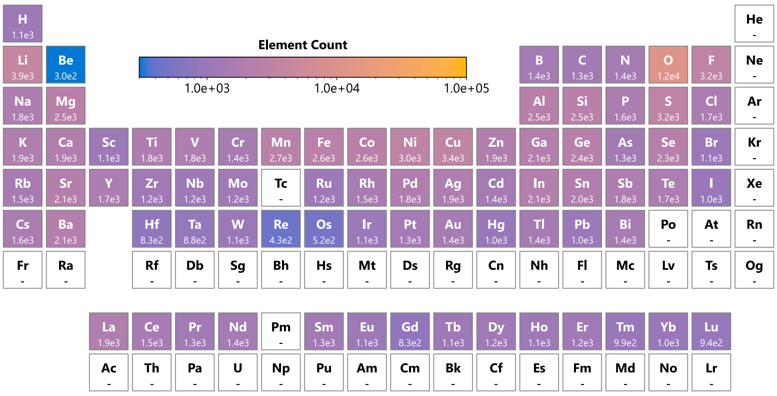

(a) MP-20

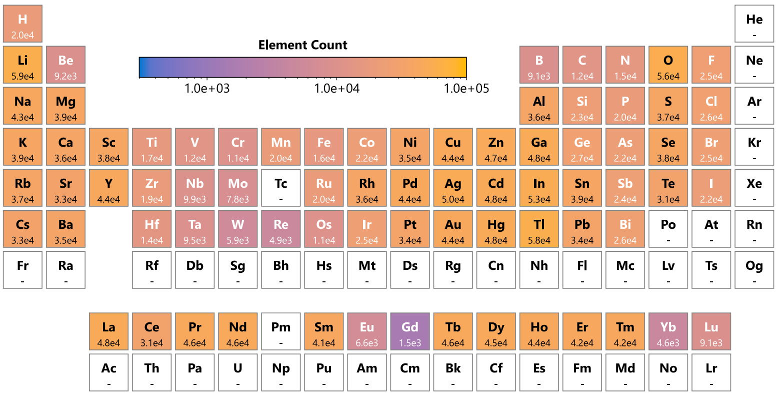

(b) Alex-MP-20

Fig. C4: Distribution of elements in MP-20 (top) and the combined Alex-MP-20 (bottom) datasets. Plot generated by pymatviz [17].

D.1.2 Hyperparameters for fine-tuning models

For fine-tuning the base model towards different properties, we used a global batch size of 128 and the Adam optimizer. Gradient clipping was applied by value at 0.5. The learning rate was initialized at 6×10^{-5} and we used the same learning rate scheduler as that for the base model. The training was stopped when the validation loss stopped improving for 100 epochs, which resulted in 32,000 - 1.1 million steps depending on the dataset.

D.1.3 MatterGen sampling parameters

For both unconditional and conditional generation, we discretized the reverse diffusion process over the continuous time interval $[0, 1]$ into $T = 1000$ steps. For each time

step, we use ancestral sampling (according to Eqs. (A6), (A8) and (A16)) to sample $(\boldsymbol{X}_{t-1}, \boldsymbol{L}_{t-1}, \boldsymbol{A}_{t-1})$ given $(\boldsymbol{X}_t, \boldsymbol{L}_t, \boldsymbol{A}_t)$ using the score model (Supplementary A.8). After each predictor step, one corrector step was applied. We used the Langevin corrector (Algorithms 4 and 5 in [35]) for the coordinates $\boldsymbol{X}_t$ and the lattice $\boldsymbol{L}_t$ with signal-to-noise ratio parameters 0.4 and 0.2, respectively.

D.1.4 Machine learning force field (MatterSim) details

We used the MatterSim [45] MLFF trained on 1.08 million crystalline structures. MatterSim is based on the M3GNet [21] architecture with three graph convolution layers, resulting in 890,000 parameters in total. To compute the energy above hull, we used the energy correction scheme compatible with MP (i.e., `MaterialsProject2020Compatibility` from `pymatgen` [15]).

D.2 Qualitative analysis of generated structures

Assessing the quality of generated crystals is difficult. In this work, we have used computational metrics to assess the quality of generated materials. However, additional human review of generated materials can be useful to identify failure modes not captured by these metrics. We perform this human analysis of generated crystals in Supplementary D.5.3, D.6.2, D.7.2, D.8.2 and D.9.2.

Ultimately, it must at least be possible to create the material in a laboratory setting for any hypothetical material to be of practical use, i.e., the material must be synthesizable. Although it is not practically possible to conclusively determine that a material is synthesizable using theoretical and computational evidence alone, we can use computation as a guide. Throughout this work we use the energy above the convex hull, calculated at 0 K under 0 GPa applied stress, as a signal [19] that a material might be stable at ambient temperature and pressure. We acknowledge that there are additional calculations we could perform (such as computing the phonon spectra) that would improve upon the approximations we make in this work, but robustly assessing synthesizability of a hypothetical crystal structure is still an open research question.

Energy above hull alone is not sufficient, since many metastable materials with finite energy above hulls ("off-hull") are routinely synthesized, while many on-hull materials have not been, despite best efforts. While an attempt [20] has been made to suggest reasonable threshold values for energy above hull, this is very dependent on chemical system: while some materials (such as carbides, nitrides) are able to tolerate very high energies above convex hull (0.1–1 eV), other materials (such as intermetallics) are only able to tolerate a very small degree of metastability (meV). It is also known [21] that traditional methods of simulation using DFT will give inaccurate energies for some materials, even after empirical corrections [13] are applied which can correct some of the better understood systematic errors. Furthermore, the energy above hull is a measure that is only meaningful if the particular chemical system has been well-explored: for unexplored or partially-explored chemical systems, the energy above hull might be inaccurate simply due to more energetically-favourable phases being unknown.

Using empirical priors to assess the synthesizability of structures is traditionally done by hand by domain experts and is therefore highly dependent on the chemical intuition and background knowledge of a particular system by the expert. It becomes more difficult for a scientist to evaluate a crystal structure picked "at random", especially as the number of elements increases, as they are less likely to have encountered this material in their prior work. Some materials are hard to analyze in this way because the prior is simply not known or may be biased; for example, any distribution of a specific property calculated from crystalline materials that have already been synthesized will include bias by not simply including the properties of materials that have not yet been synthesized. These biases can be because certain elements are more abundant, cheaper, or easier to process, or because certain materials have garnered more technological interest, rather than because of an *a priori* physical reason why those materials could not be made. Simply put, we do not yet know what the distribution of "possible" crystal structures looks like, even within certain constraints (e.g., maximum primitive cell size or number of elements). Therefore, assessing synthesizability using empirical priors is difficult, but might still provide insights. When examining a generated material, in addition to performing a literature review, additional factors can be considered, including:

1. Symmetry; crystals are defined by their symmetry, and nature prefers symmetrical crystals unless there is a specific mechanism by which symmetry is broken. While lower symmetry would be expected as the number of elements in a system increases, in general generated crystals are expected to be symmetrical. P1 crystals are rare in nature, and when they are reported in databases this is often either because they have not been refined or even because of mis-identification [22].
2. The presence of defects. Defects could include structural distortions, with a crystal containing the "correct" atoms but in distorted geometry, or could include point defects, such as vacancies or interstitial atoms. The presence of defects is not necessarily bad; for example, many "off-stochiometric" materials such as NiO_x are routinely synthesized which can contain large concentrations of vacancies. Sometimes vacancies might be required so that a structure might charge balance; a classic example might be the bixbyite crystal structure derived from a fluorite structure with an ordered array of structural vacancies. However, the presence of defects could also be a concern, especially in the case of unexplored chemical systems where a material might be erroneously calculated to be "on hull" due to incomplete knowledge of that chemical system.
3. Local atomic environments should be reasonable, meaning that the material contains reasonable bond lengths and co-ordination polyhedra, where other materials with those combinations of elements are known.
4. The material should charge balance if highly ionic, meaning that the sum of the formal valence of its constituent atoms is zero. If a material does not charge balance, and is ionic, it will likely have a very low defect formation energy if synthesizable. However, the importance of charge balance should be taken with caution, since the proportion of new materials that have been discovered that are nominally charge-balanced has decreased over time [23], with only around 40% newly-discovered

materials being charge-balanced compared to over 80% of materials discovered a hundred years ago.

The definition of a material used in this work (Section 2) also allows for many potential failure modes including but not limited to: non 3D periodic materials such as 2D materials that contain a vacuum in one dimension, amorphous materials, or other random arrangements of "atoms-in-a-box". Effort can be made to avoid these classes of materials by altering the training data of the generative model. Some efforts have been made to algorithmically categorize crystals [24], which could then be used to this effect, however these tools are not yet well-developed, and some spurious structures in the training set should be expected.

Finally, we highlight that MatterGen can generate only ordered materials, whereas many real materials exist as alloys or solid solutions. In traditional DFT similar constraints apply, where only fully ordered materials can be calculated (meaning, a material that contains whole atoms, with exactly one atom on a given atomic site). However, real materials are often disordered, with fractional atomic occupancy (on *average*, a given atomic site might contain, say, 50% of one atom, and 50% of another atom). A disordered material has many "ordered approximations"–small unit cells with the correct overall composition–that can represent the parent disordered material. Nonetheless, the model can generate structures that are ordered approximations of a disordered material. As such, MatterGen might generate several ordered approximations of the same parent disordered material. We take this possibility into account when computing novelty and uniqueness by virtue of our *ordered-disordered* structure matcher (Supplementary D.4). Therefore, whenever the model generates ordered approximations of known disordered materials, these should be correctly identified as non-novel. The same applies to ensembles of ordered approximations of the same disordered material, which should be correctly identified as non-unique.

Given these factors, we acknowledge that there are limitations in materials discovery efforts that still require methods advancements to overcome. In some cases, we have demonstrated that our model is able to generate synthesizable materials by either synthesizing a material directly or by re-discovering previously synthesized materials (e.g., as in Section 2.6). In other cases, we restrict our confidence that structures we generate are synthesizable to the level of theory and computation we use in this work, in addition to the finite reference we use for the convex hull. We have attempted human-assisted evaluation of predicted structures using empirical priors to gain additional knowledge for how our model performs at generating synthesizable materials.

D.2.1 Visualization

Manual analysis of crystal structures can be influenced by how they are represented visually, for example the specific bonds that are drawn. In this work, crystal structures are visualized using Crystal Toolkit [25] with the CrystalNN [26] bonding algorithm, since this is known to give good results in most cases. A uniform atomic radius was used since a wide variety of chemical bonding is expected to be present, and no one type of atomic radii (covalent, ionic, etc.) can be assumed. When valences are indicated, these are formal valences assigned using heuristic methods in `pymatgen` [15]. With the

exception of Fig. 1, all visualizations are of a single cell and all atoms in neighbouring cells that form a bond with atoms in the cell. All visualizations of crystal structures are in their conventional unit cell, then manually rotated to improve clarity.

D.3 Stability, uniqueness and novelty (S.U.N.)

Throughout the manuscript, we evaluate the performance of MatterGen and other methods by measuring the fraction of structures that are *stable*, *unique*, and *novel* (S.U.N.). Below, we provide detailed definitions of these three criteria.

D.3.1 Stability

We consider a structure to be stable if its energy per atom after relaxation via DFT is within 0.1 eV/atom above the convex hull defined by our Alex-MP-ICSD reference dataset comprising 850,384 unique, ordered structures recomputed from the MP, Alexandria, and ICSD datasets. We stress that stability is always computed using DFT and against a DFT-computed convex hull; MLFF-predicted energies are only used for intermediate filtering steps in some computational experiments. If we generate multiple compounds within the same convex hull diagram whose energies per atom are below the stability threshold, we consider all of them to be stable, irrespective of whether all structures would still be below the threshold when considering the combined convex hull. The only exception where we indeed consider the combined convex hull is in Fig. 3(c-d), where we generate materials given a target chemical system. That is because we explicitly define the task as finding the most stable structures in a given chemical system.

We adopt a threshold of 0.1 eV/atom to determine stability as Aykol et al. [20] have shown that the amorphous limit for meta-stability is between 0.05 eV/atom and 0.9 eV/atom depending on the chemical system. Our threshold ensures that enough metastable materials are covered but we do not include too many unsynthesizable materials. Note also that our experimentally synthesized structure (Section 2.6) is metastable at 0.024 eV/atom above the convex hull and would have been a missed synthesizable sample at a strict 0.0 eV/atom cutoff. A consequence of this choice is that it is less likely for our model to generate novel structures exactly on the convex hull, e.g., when we condition on chemical system (Fig. 3(c-d)). We briefly discuss results for a 0 eV/atom cutoff in Supplementary D.6.3.

D.3.2 Uniqueness

When we use a method like MatterGen to propose a batch of material candidates, we would like the candidates to be diverse. Since diversity is difficult to measure, we instead resort to measuring whether the candidates are *unique*. We consider a structure to be unique if it does not match any other structure in a batch of samples generated by a given method, where uniqueness is computed among all samples in a batch with the same reduced chemical formula via our *ordered-disordered* structure matcher (Supplementary D.4). Since we do not require two structures to have the same assigned space group for them to match, it is possible (albeit very rare) that two structures nominally belonging to different space groups are matched.

Note that the fraction of unique structures is a function of the number of generated samples (see Fig. 2(d)). Throughout the manuscript, we therefore only evaluate uniqueness between methods on the same number of samples, e.g., 100 samples per chemical system in Section 2.3.

D.3.3 Novelty

We consider a structure to be novel if it does not match any structure in an extended version of our Alex-MP-ICSD reference dataset, containing 117,652 disordered ICSD structures in addition to the 850,384 ordered structures used to compute the reference convex hull (Fig. C3). To compute novelty, we first compare each generated structure with all other *ordered* structures from our reference dataset Alex-MP-ICSD with the same reduced formula. Additionally, we compare generated structures with all other *disordered* structures from our extended reference dataset that have a similar reduced formula. In particular, we allow for small differences in the reduced formula (relative proportion of each element within 10%) in order to account for disordered structures with small variations in the element proportion. For example, we attempt to match an ordered structure with reduced formula $Fe_2Ni_3O_5$ with a disordered ICSD structure with reduced formula $Fe_{2.2}Ni_{2.8}O_5$. Similar to our uniqueness calculations (Supplementary D.3.2), we employ our *ordered-disordered* structure matcher (Supplementary D.4) to assess whether two structures match. Since we do not require two structures to have the same assigned space group for them to match, it is rare but possible that two structures nominally belonging to different space groups are matched.

Note the difference between uniqueness and novelty: uniqueness refers to whether a generated structure is distinct from all other generated structures by the same method; in contrast, novelty measures whether a generated structure is distinct from all other structures in our extended reference dataset. For example, when asked to generate N structures, a model might generate a previously unknown structure N times, in which case novelty would be high but uniqueness would be low. A practically useful model should be able to generate both unique and novel structures.

D.4 Ordered-disordered structure matcher

As recently highlighted by Leeman et al. [40], an important step in high-throughput materials discovery is to check whether a predicted structure matches another (already known) structure when considering compositional disorder. However, identifying whether two ordered crystal structures represent the same material while accounting for the permutation of chemically similar elements is non-trivial. The `pymatgen StructureMatcher` is one of the most widely-employed computational tools to assess whether two crystal structures with the same chemical composition are the same. Yet, it fails to match pairs of structures where elements often found in solid substitution [27] (e.g., Au and Ag) are residing in different non-symmetrically-equivalent sites. This results in inflated uniqueness and novelty metrics since structures that are different ordered approximations of the same compositionally disordered material are not identified as such.

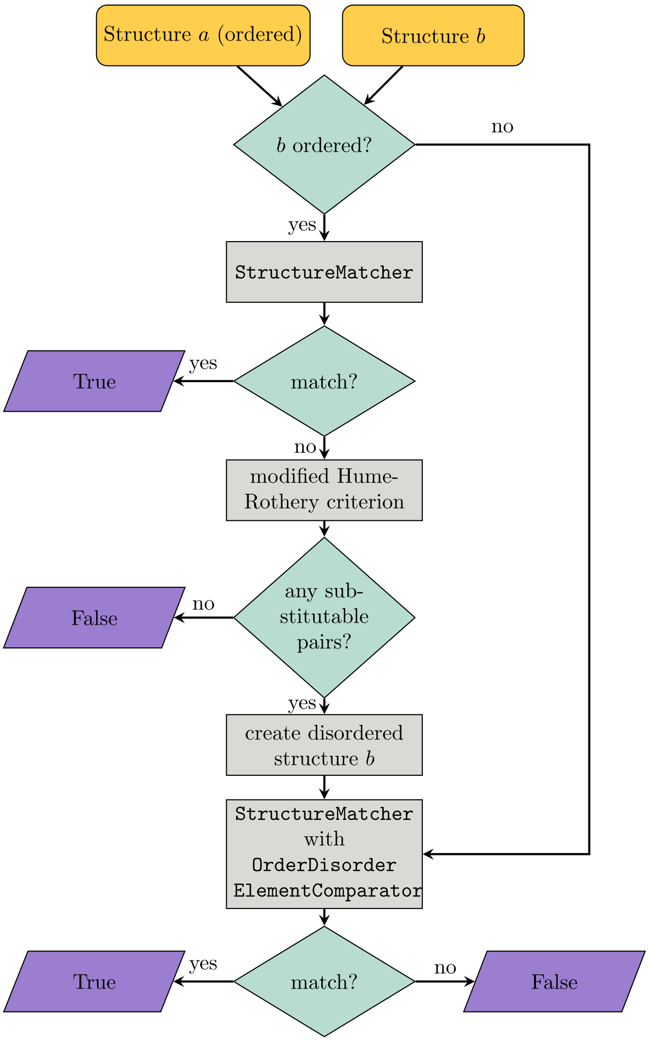

Fig. D5: Flow chart for the proposed *ordered-disordered* structure matcher when comparing two structures, assuming structure a is ordered and both structures contain the same set of elements.

To overcome this issue, we develop an *ordered-disordered* structure matcher algorithm to assess whether a pair of structures can be identified as ordered approximations of the same underlying compositionally disordered structure or, similarly, whether an ordered structure can be identified as an ordered approximation of a known compositionally disordered structure. We design this structure matcher as an extension of the `StructureMatcher` class in `pymatgen` that can match a disordered structure to one of its ordered approximations (Fig. D5). The *ordered-disordered* structure matcher is

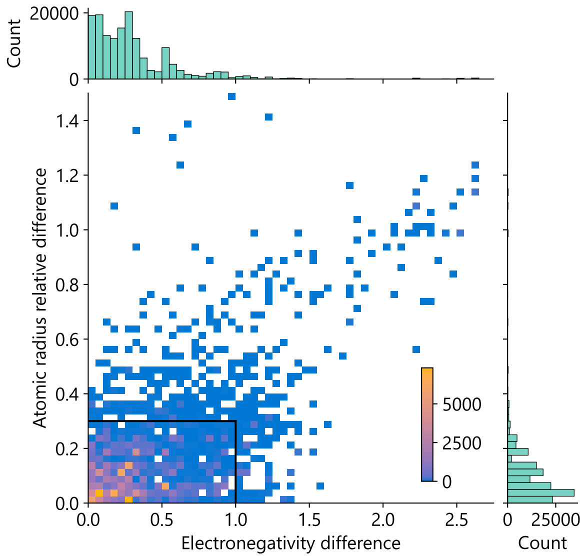

Fig. D6: Occurrence of element substitution pairs in compositionally disordered structures within the ICSD dataset, as a function of electronegativity difference and atomic radius relative difference. The black lines indicate the threshold for the modified Hume-Rothery criterion outlined in Eq. (D48); 95% of all element substitution pairs fall within its bounds.

strictly more generous in matching structures than the `pymatgen StructureMatcher`; therefore, novelty and uniqueness values computed with this new matcher will be strictly equal or lower than the ones computed with the standard `StructureMatcher` (Supplementary D.4.1).

When comparing two ordered structures (with the same set of elements), the *ordered-disordered* structure matcher will first try to match them using the `pymatgen StructureMatcher`. If the structures match, the algorithm terminates and returns `True`. If no match is found, the algorithm applies the following criterion inspired by the well-known Hume-Rothery rules [27] to identify whether pairs of elements a and b could substitute each other:

$$|\chi_a - \chi_b| \leq 1 \quad \text{and} \quad \frac{2|r_a - r_b|}{r_a + r_b} < 0.3, \tag{D48}$$

where χ_a indicates the electronegativity of element a and r_a its atomic radius. The threshold values of 1 and 0.3 were chosen by looking at pairs of elements that appear in partial occupancy sites within the database of disordered ICSD structures (Fig. D6). If no pair of substituting elements can be found, the algorithm returns `False`, since neither of the structures could be thought of as an ordered approximation of a compositionally disordered structure. If, instead, two or more pairs of elements pass the modified Hume-Rothery criterion in Eq. (D48), then one of the

two ordered structures is transformed into its disordered counterpart, where every clique of elements that could substitute each other is given proportional occupancy in the disordered sites originally belonging to one of those elements. Finally, we employ a `StructureMatcher` with `attempt_supercell=True`, `allow_subset=True`, and `comparator=OrderDisorderElementComparator` to verify whether one ordered structure matches the disordered variant of the other structure. In cases where we want to directly compare an ordered structure with a compositionally disordered structure (e.g., when computing novelty w.r.t. disordered ICSD structures), then only the final step of the above algorithm is executed.

Note that the current implementation of the *ordered-disordered* structure matcher attempts to match pairs of *ordered* structures only if their reduced formulas match, i.e., they contain the same elements in the same ratio. For example, matching will be attempted for two ordered structures with formulas $Fe_2Ni_4O_6$ and $FeNi_2O_3$, but will not attempted for two ordered structures with formulas $Fe_2Ni_4O_6$ and $Fe_1Ni_5O_6$. Whether two crystalline materials with different proportions of elements that are known to form solid substitutions should be considered distinct or not is an open question; here, we adopt the former definition.

Benchmark

To verify the performance of the *ordered-disordered* structure matcher, we generate a dataset of 24,256 ordered approximations of 1,196 disordered alloy structures (1,164 distinct reduced formulas) using the `pymatgen` `EnumerateStructureTransformation` class. We then compute precision and recall of the *ordered-disordered* structure matcher on this paired dataset where many ordered structures match to each compositionally disordered structure. We obtain 100% recall and 100% precision, meaning that the *ordered-disordered* structure matcher correctly matches ordered structures and the disordered structure they were generated from, while it never matches an ordered structure with a disordered structure it was not generated from. However, we note that the precision measured here could be inflated since only 32 out of 1,196 disordered structures share the same reduced formula.

D.4.1 MatterGen performance difference between standard and *ordered-disordered* structure matcher

In this section, we compare the difference in uniqueness, novelty, and S.U.N. metrics when using the commonly-employed `pymatgen` `StructureMatcher` instead of our *ordered-disordered* structure matcher used throughout this work. Metrics computed using the `pymatgen` `StructureMatcher` employ the following parameters: `ltol`=0.2, `stol`=0.3, `angle_tol`=5, which are the defaults found in `emmet` [12]. As shown in Table D3, the *ordered-disordered* structure matcher identifies a strictly higher number of matches between structures, thus resulting in strictly lower uniqueness, novelty, and S.U.N. values. Additionally, the *ordered-disordered* structure matcher allows matches between generated ordered structures and disordered ICSD structures, which is not possible with the commonly-employed `pymatgen` `StructureMatcher`, thus further reducing uniqueness and novelty metrics. While we only report differences for MatterGen metrics appearing in the main text for conciseness, we note that novelty,

uniqueness, and S.U.N. metrics decrease for all methods and benchmarks when using the *ordered-disordered* structure matcher instead of the `pymatgen` `StructureMatcher`.

Metric	Appearing in	`pymatgen` `StructureMatcher`	*ordered-disordered* structure matcher
% Novelty (1 million samples)	Fig. 2d	68 %	61 %
% Uniqueness (1 million samples)	Fig. 2d	86 %	80 %
% S.U.N. (Alex-MP)	Fig. 2e (teal)	44 %	39 %
% S.U.N. (MP)	Fig. 2e (purple)	24 %	22 %
% S.U.N. (well explored)	Fig. 3a (teal)	85 %	84 %
% S.U.N. (partially explored)	Fig. 3a (teal)	79 %	65 %
% S.U.N. (not explored)	Fig. 3a (teal)	54 %	49 %
% S.U.N. (3 elements)	Fig. 3b (teal)	86 %	77 %
% S.U.N. (4 elements)	Fig. 3b (teal)	73 %	65 %
% S.U.N. (5 elements)	Fig. 3b (teal)	60 %	54 %
# S.U.N. & mag. den. > 0.2 Å^{-3}	Fig. 4g (teal)	27	18
# S.U.N. & bulk mod. > 400 GPa	Fig. 4h (teal)	114	106
# S.U.N. (mag. density)	Fig. 5a (yellow)	248	59
# S.U.N. (mag. density & HHI)	Fig. 5a (teal)	107	49

Table D3: Novelty and uniqueness metrics for MatterGen appearing in the main text computed using the default `pymatgen` `StructureMatcher` and our novel *ordered-disordered* structure matcher used throughout this work.

D.5 Generating stable and diverse materials

This section provides supplementary information to the results in Section 2.2.

D.5.1 Additional experimental details

For the results of DiffCSP, we use the checkpoint released by the authors, and for all other baselines we randomly subsample the 10,000 structures used in [4]. To evaluate the performance of a generative model on the task of unconditional generation, we look at two keys metrics. First, we use the RMSD between the generated and the DFT-relaxed structure to measure local stability. Second, we use the fraction of S.U.N. structures to capture global stability and, to some extent, diversity (Supplementary D.3). The RMSD metric is defined as

$$RMSD = \sqrt{\min_{\boldsymbol{P}} \frac{1}{N} \sum_{n}^{N} \left| \tilde{\boldsymbol{x}}^{gen}_{\boldsymbol{P}(n)} - \tilde{\boldsymbol{x}}^{DFT}_{n} \right|^2}, \qquad \text{(D49)}$$

where $\tilde{\boldsymbol{x}}_n$ indicates the Cartesian coordinates of atom n, and $\boldsymbol{P}$ is the element-aware permutation operator on the atoms of the generated structure. A lower RMSD indicates that generated structures are closer to their DFT-relaxed counterpart. This in turn reduces the computational time for the DFT relaxation, which is typically the most costly part of crystal structure generation.

Model	Avg. RMSD [Å]	% S.U.N.
MatterGen (Alex-MP)	**0.021**	**38.57**
DiffCSP (Alex-MP)	0.104	33.27

Table D4: Results of training the DiffCSP baseline on our Alex-MP dataset. Best result highlighted in bold.

The plots displayed in Fig. 2(b,c,e,f), and the structures shown in Fig. 2(a) refer to samples of 1024 generated structures that have also been relaxed via DFT.

D.5.2 Additional baseline comparison

In Table D4 we present an additional result comparing to the DiffCSP baseline. Here, we have trained the baseline on the same Alex-MP dataset as MatterGen. We find that DiffCSP's generated structures are roughly 5x farther from their DFT-relaxed local optima, and MatterGen produces stable, novel, and unique structures at a more than 15% higher rate than DiffCSP.

D.5.3 Additional qualitative analysis of structures

Within the 1024 structures generated unconditionally for Fig. 2(a), a total of 17 unique, on-hull crystal structures were found: 7 binaries, 7 ternaries, and 3 quaternaries (Table D5). Of these, three had P1 symmetry, and one contained molecules or was a molecular crystal. Prototype assignment limited to prototypes available in the `robocrystallographer` [28] tool, which will assign the "closest" matching prototype subject to tolerances. The ability for a composition to charge balance assessed by `pymatgen` and known common oxidation states for each element. As previously discussed, a generated crystal can still be reasonable even if it does not charge balance, and not all materials are ionic.

Four randomly-selected examples from the full set of unconditional structures (both novel and not novel) were highlighted in Fig. 2 in the main text. These were $BaLa_2Ir$, K_3AlCl_6, $NaNiIO_6$, and $NaSm(TmTe_2)_2$. For $BaLa_2Ir$, it is well-known that La_2Ir forms an intermetallic with a Laves structure, and that Ba can often substitute for La since both can exist in a +2 oxidation state with a 6s2 outer shell. However, in this example, we see octahedrally co-ordinated Ir bonded to La, with Ba inserted as a single plane of atoms in a close-packed configuration, as it would exist in elemental Ba. It is unclear if this material could exist. The K_3AlCl_6 has low space group symmetry ($P\bar{1}$), but consists of Al in an ideal octahedral co-ordination, with K in a mixed bonding environment. This structure charge balances under the assumption of Al^{3+}, K^+ and Cl^-, and could be thought of as derived from a defected rocksalt. The $NaNiIO_6$ structure exists in the training set and is well-known belonging to a family of periodate structures $AMIO_6$ (where A is an alkali metal and M is another metal atom). $NaSm(TmTe_2)_2$ is a rocksalt structure with Te in the anion site and a mix of Na, Sm and Tm in the cation sites.

Formula	Symmetry	# Elements	Charge balances	Prototype	Classification
Ba_4Au	C2/m	2	-	-	Crystal with 1D chains
SbAu	$P6_3/mmc$	2	Yes	Molybdenum Carbide MAX Phase	Bulk crystal
SeS	P1	2	Yes	red selenium	Crystal with 1D chains
V_3Cl_8	C2/m	2	Yes	-	Layered/2D crystal
KCl_8	P1	2	-	-	Hybrid
AlI_7	P1	2	-	-	Crystal with 1D chains
VBr_5	Cm	2	Yes	Silicon tetrafluoride	Molecular crystal
GdScBi	P4/nmm	3	-	Matlockite	Bulk crystal
$Sm(FeC)_2$	Fddd	3	Yes	-	Bulk crystal
Tl_4IrO_6	C2/m	3	Yes	-	Bulk crystal
$CsTe_2Pd$	C2/m	3	Yes	-	Bulk crystal
Sb_5PPb_2	Amm2	3	Yes	-	Bulk crystal
$TePb_5Cl_8$	C2/m	3	Yes	-	Bulk crystal
$FeCoH_2$	P4/mmm	3	-	Caswellsilverite	Bulk crystal
$TbCe(Ho_2Te_3)_2$	Cm	4	-	alpha Po	Bulk crystal
Zn_2Ni_6BH	$Fm\bar{3}m$	4	-	(Cubic) Perovskite	Bulk crystal
Te_2MoWSe_2	Cm	4	Yes	Molybdenite	Layered/2D crystal

Table D5: Summary information on 17 crystal structures that were calculated as being novel, unique, and thermodynamically stable from a batch of 1024 crystal structures from an unconditional generation task.

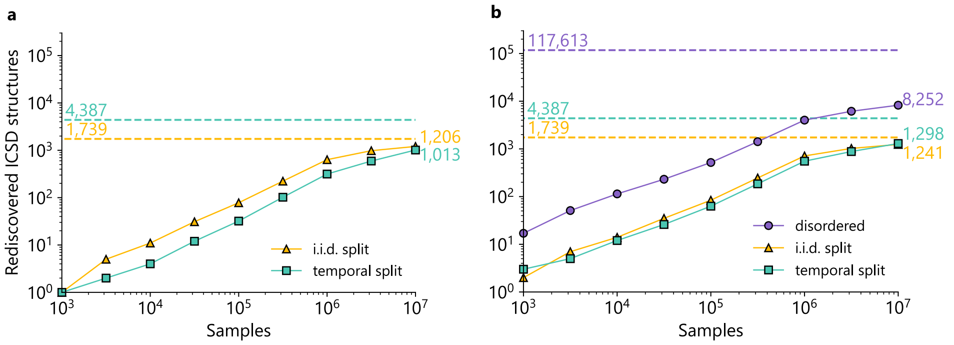

Fig. D7: Rediscovery of ICSD structures with **(a)** default `pymatgen` structure matcher, and **(b)** *ordered-disordered* structure matcher. Dashed lines indicate the total number of ICSD structures in each split.

D.5.4 Rediscovering experimental materials

Experimental verification is the ultimate test of synthesizability for any generative model for materials. In addition to the experimental results in Section 2.6, we can evaluate whether MatterGen generates reasonable materials which could be synthesized by trying to 'rediscover' experimentally known materials which the model has not seen during training [23]. For this, we generate 10 million samples using MatterGen, and check whether they match experimental materials from a hold-out set. As mentioned in Supplementary C.1, we hold out 4,387 experimental structures from ICSD for testing purposes. These are experimental ICSD structures which were not in the Materials Project database at our cutoff date of July 5, 2022. Our cutoff date for including structures from ICSD is July 13, 2023. In addition, we have 1,739 ICSD structures held out of the training dataset via our i.i.d. splitting between training and validation set.

We refer to these two sets of held-out experimental ICSD structures as the ‘temporal’ and ‘i.i.d.’ splits, respectively.

In Fig. D7(a) we see that MatterGen successfully re-discovered more than 2,200 experimental ICSD structures, of which 1,206 were from the i.i.d. split and 1,013 from the temporal split. Here, we consider two structures to be equal if they match using the *ordered* version of the `pymatgen StructureMatcher` (Supplementary D.4.1). This imposes a stricter matching criterion—and therefore more conservative rediscovery rates—than the *ordered-disordered* structure matcher (Fig. D5) used throughout this work. Additionally, we employ the *ordered-disordered* structure matcher to evaluate rediscovery of the ca. 118k disordered ICSD structures from our extended reference dataset, which have not been seen by the model.

In Fig. D7 we show that, besides slightly higher rediscovery rates for the ordered ICSD hold-out structures, MatterGen’s generated structures also match around 8k experimentally confirmed disordered ICSD structures. Here we filter out any structures which match to a training structure using the *ordered-disordered* structure matcher, which ensures that ‘rediscovered’ structures are not merely training structures modified according to the criteria of the *ordered-disordered* structure matcher.

These results demonstrate that MatterGen is able to generate *synthesizable* structures outside its training data. This suggests that MatterGen can be used to propose novel structure candidates which can subsequently be synthesized.

D.6 Generating materials with target chemistry

This section provides supplementary information to the results in Section 2.3.

D.6.1 Additional experimental details

We explore the capability of MatterGen to find novel stable crystals across the 27 chemical systems listed in Table D6. We group the systems in terms of how many elements they contain (ternary, quaternary, and quinary), and in terms of how many structures near the the convex hull were present in the reference Alex-MP-ICSD dataset (‘well explored’, ‘partially explored’, ‘not explored’). The latter classes are defined as follows:

- ‘well explored’: systems with the highest numbers of structures near the convex hull. We removed all structures belonging to ‘well explored’ systems from the training data set to assess the capability of our model to recover existing stable structures without having seen them during training.
- ‘partially explored‘: systems that lie between the 30th and the 90th percentile of the distribution of chemical systems based on the number of structures they have near the convex hull. This class was designed to assess the capability of our model to expand known convex hulls. Therefore, we did not remove the existing data belonging to such systems from the training set.
- ‘not explored’: systems with no data near the convex hull. This class was designed to test our model in chemical systems where no structures on the hull are present in the reference dataset.

Here, we define 'near the convex hull' structures as structures whose energy per atom is between 0.0 and 0.1 eV/atom above the convex hull. For all three groups, we randomly chose nine ternary, nine quaternary and nine quinary chemical systems (Table D6). Moreover, we replaced those chemical systems that had an overlap of more than two elements with another system to promote chemical diversity. The replacement was chosen randomly as well.

For this task, we fine-tune our base model on two properties: chemical system and energy above hull. We encode the latent embedding for the energy above hull and the chemical system as detailed in Supplementary B.2.1 and Supplementary B.2.2, respectively. Both properties are available for all structures in the training set of the base model. Therefore, the training set is used in full for fine-tuning. At sampling time, we condition on both an energy above the convex hull of 0.0 eV/atom, and on the chemical system we want to sample.

To compare the performance of MatterGen against substitution and RSS, we use the MatterSim MLFF [45] (Supplementary D.1.4) to relax the generated structures, and then perform *ab initio* relaxation and static calculations via DFT (Supplementary C.2). In particular, we perform the following steps: (1) generate structures, (2) relax structures using MatterSim, (3) filter structures for uniqueness, (4) select the 100 structures with lowest predicted energy above hull according to MatterSim, (5) run DFT on these structures. We report metrics only with respect to those structures. To allow for a fair comparison between our generative model and non-generative approaches, we employ MLFF relaxation on a greater number of samples for the latter. For RSS, we sample 600,000 structures per chemical system according to the protocol described in Supplementary D.6.1. For substitution, we enumerate every possible structure according to the algorithm detailed in Supplementary D.6.1, which yields between 15,000 and 70,000 structures per chemical system. For MatterGen, we generate 10,240 structures per chemical system.

	**Ternary**	**Quaternary**	**Quinary**
**Well explored**	O-Sr-V	Bi-Cu-Pb-S	C-H-N-O-S
	Mn-O-Se	As-Cl-O-Pb	Ba-Ca-Cu-O-Tl
	La-Mo-O	Fe-Na-O-P	Eu-F-K-O-Si
**Partially explored**	Li-Pr-Te	Cl-Cu-Dy-Rb	Cu-Gd-O-Ru-Sr
	C-Pr-Ru	Na-Te-Tm-Zr	La-Na-O-Sb-Sc
	C-Mg-Sc	F-Mg-Rb-Sn	Cs-F-O-Tl-Zr
**Not explored**	Br-Pb-Rh	Cr-Ga-Mg-P	Al-C-H-Sb-Zr
	As-Cu-Sr	C-Cl-Ho-Ru	As-Br-Cr-I-Pt
	Cl-Er-In	Al-Au-Co-S	K-Mo-O-P-Sr

Table D6: Categorization of the 27 chemical systems used to benchmark model capabilities on chemical system exploration

Random structure search details

For every chemical system, we performed two rounds of RSS using the `airss` [43] package. In each round, we generated 300,000 structures by sampling 100,000 structures

across three different ranges of number of atoms per unit cell. We used the following non-overlapping intervals: 3-9, 10-15, and 16-20 for ternary systems; 4-10, 11-15, and 16-20 for quaternary systems; 5-11, 12-16, and 17-20 for quinary systems. For the first round, we used `airss` to propose structures without structural relaxation using `MINSEP = 0.7-3` (minimum separation between atoms in Å) and `SYMMOPS = 2-4` (number of symmetry operations). All proposed structures were relaxed using the MatterSim [45] MLFF (Supplementary D.1.4), and the resulting 300,000 MLFF relaxation trajectories were used in the second round of RSS to automatically tune the `MINSEP` parameter. Again, `airss` was run without structural relaxation followed by a MLFF relaxation. Finally, we combined the 600,000 MLFF-relaxed structures from both rounds and ran DFT structural relaxation and static calculation on the 100 unique structures with the lowest predicted energy above hull according to MatterSim.

Substitution details

A total of 5,143 ordered crystal structures (2,695 ternary, 1,875 quaternary, and 573 quinary) with less than 100 atoms in a unit cell from the ICSD [39] were used as prototypes. For each chemical system in Table D6, we computed all possible unique substitutions of the prototypes, relaxed all structures using the MatterSim [45] MLFF (Supplementary D.1.4), and selected the 100 unique structures with the lowest predicted energy above the hull according to the MLFF. Finally, we ran DFT structural relaxation and static calculation on the selected structures.

D.6.2 Additional qualitative analysis of structures

The V-Sr-O chemical system example provided in Fig. 3 produced three novel on-hull crystal structures discovered by MatterGen: SrV_2O_6 (V^{5+}), $Sr_3(VO_4)_2$ (V^{5+}) and SrV_2O_4 (V^{5+}). This chemical system has been well-studied in literature and also contains $SrVO_3$, a well-known perovskite [29], $Sr_3(VO_4)_2$ is expected to be synthesized in a cation-deficient variant of the $SrVO_3$ crystal structure [30]. We also note that MatterGen re-discovers the Sr_2VO_4 structure, which was already known but not included in the training dataset.

Vanadates are known to by synthesizable in a variety of frameworks, with the expected co-ordination of the VO_4 sub-unit varying with oxidation state [31] from the ideal tetrahedron in V^{5+} to a variety of other co-ordination environments. All generated structures have plausible atomic environments with VO_4 sub-units, either ideal or distorted, and oxygen co-ordinated Sr atoms.

One SrV_2O_4 structure, having C2/m symmetry, consists of a layers of ideal VO_4 edge-sharing tetrahedra separated by a Sr in a triangular prismatic bonding configuration, resulting in a 1D channel of voids in the Sr layer.

D.6.3 Generating structures at 0 eV/atom of convex hull

In Section 2.2 and Table D5, we show that MatterGen generates around 3 % structures stable at 0 eV/atom of the Alex-MP-ICSD convex hull. This result is comparable with the training data distribution, where only 8.0 % structures in the Alex-MP-ICSD are stable at 0 eV/atom of the convex hull. The fraction of S.U.N. materials at the

Chemical System	MatterGen conditioned on E_{hull}	MatterGen
Cl-Er-In	40	27
As-Cl-O-Pb	29	44
O-Sr-V	11	9
Mn-O-Se	10	6
Br-Pb-Rh	10	3
Eu-F-K-O-Si	9	0
C-Pr-Ru	7	13
C-H-N-O-S	2	0
C-Mg-Sc	2	6
Al-Au-Co-S	2	2
La-Mo-O	2	1
Li-Pr-Te	1	0
Cl-Cu-Dy-Rb	1	0
Cs-F-O-Tl-Zr	1	0
Bi-Cu-Pb-S	0	0
Al-C-H-Sb-Zr	0	0
As-Br-Cr-I-Pt	0	0
As-Cu-Sr	0	0
Ba-Ca-Cu-O-Tl	0	0
Cu-Gd-O-Ru-Sr	0	0
K-Mo-O-P-Sr	0	0
Fe-Na-O-P	0	0
F-Mg-Rb-Sn	0	0
La-Na-O-Sb-Sc	0	0
Na-Te-Tm-Zr	0	0
Cr-Ga-Mg-P	0	0
C-Cl-Ho-Ru	0	0
Total	127	111

Table D7: Percentage of S.U.N. structures where the stability threshold has been set to 10^{-5} eV/atom. The middle column reports performance for a model conditioned on both chemical system and $E_{\text{hull}} = 0.0$ eV/atom, the right column for a model conditioned only on chemical system.

0 eV/atom threshold is 1.7 %, where 59.4 % of structures are novel and 100 % are unique, similar to our main result using 0.1 eV/atom as the stability threshold (see Supplementary D.3 on why we use a 0.1eV/atom threshold).

We can encourage our model to generate structures on the convex hull by explicitly conditioning on the energy above the hull values. As an example, in Table D7, we show that we can improve the S.U.N. at 0 eV/atom above the hull by jointly conditioning on chemical system and $E_{\text{hull}} = 0$ eV/atom for most chemical systems. Additionally, the energy above the hull conditioning doubles the total number of structures on the combined hull found by MatterGen on the 27 chemical systems we consider (from 36 to 78). We note that Section 2.3 reports results for the model jointly conditioned on chemical system and energy above the hull.

D.7 Designing materials with target symmetry

The symmetry of a material directly affects its electronic and vibrational properties, and is a determining factor for its topological [32] and ferroelectric [33] characteristics. The generation of S.U.N. materials with a given symmetry is a challenging task, as the symmetric arrangement of atoms in space is hard to enforce without resorting to explicit constraints based on already known materials. That is because the space group of a structure might be sensitive to small variations in the atomic positions, e.g., this is different from image generation problems where small differences in pixel space do not change the class/concept of the output.

Here, we assess MatterGen's ability to generate S.U.N. materials with a target symmetry by fine-tuning it on space group labels. We choose 14 space groups at random from the subset of space groups that had at least 1000 entries in the training dataset, two for each of the seven crystal systems. We generate 256 structures per space group and relax them with DFT.

The results are shown in Fig. D8(a). On average, the fraction of generated structures with the target space group is 53% (teal), 39% if we also filter for stability (yellow), and 16% if we additionally filter for novelty and uniqueness (purple). We note some interesting differences between the 14 space groups. MatterGen has a very high success rate in sampling stable structures that belong to the highly-symmetric F$\bar{4}$3m space group (61%), but these structures have low novelty and/or uniqueness. This can be explained by the low number of degrees of freedom for structures in this space group: only four distinct Wyckoff positions having less than 16 sites are present, all with no spatial degree of freedom. This implies that structures with fewer than 20 atoms within the F$\bar{4}$3m space group have mostly compositional degrees of freedom. Moreover, many structures with this space group are present in the reference data set (32,422 structures); it is therefore hard for the model to generate novel samples. On the other hand, the model has more difficulty in sampling stable structures belonging to low-symmetry space groups such as P$\bar{1}$, P1m1, or C12/c1, but most of those structures are novel and unique, possibly due to the high number of structural degrees of freedom present. Finally, we note that some of the most highly symmetric space groups that were chosen, e.g., P$\bar{6}$m2 and Im$\bar{3}$ achieve a success rate of being in the target space group and of being S.U.N. of at least 10% . This is a notable result given that most previous generative models struggled in generating highly symmetric crystals [4, 12]. In Fig. D8(b), we show 14 randomly generated S.U.N. structures, one from each space group in Fig. D8(a). A brief qualitative analysis of the displayed structures can be found in Supplementary D.7.2

In Fig. D9 we compare the marginal distribution of space groups of the training data (purple) with the space group of samples generated using MatterGen and relaxed with DFT (teal), where target space groups are drawn from the training data distribution along with the number of atoms in the unit cell. We display only space groups with at least 1000 entries in the training dataset for legibility. We observe that structures sampled by the symmetry-conditioned MatterGen model mostly follow the space group distribution of the training data, with the exception of the P1, P1m1, and C1m1 space groups, which are over-sampled.

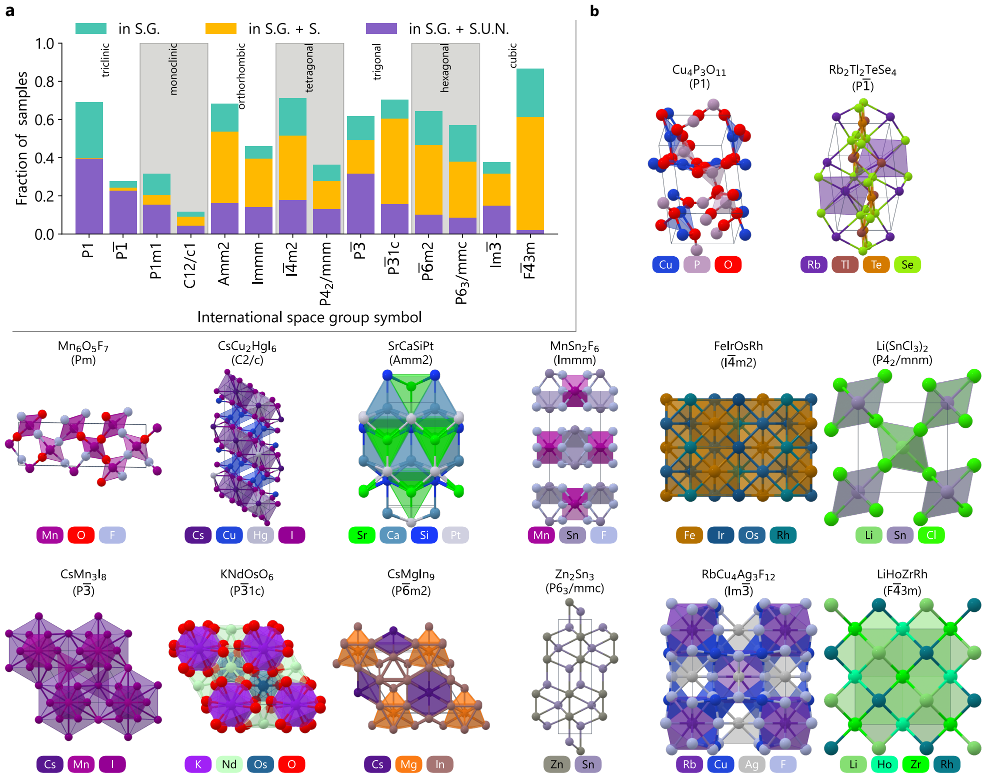

Fig. D8: **Generating materials with target symmetry.** **(a)** Fraction of generated structures that belong to the target space group (teal), are also stable (yellow), and are also S.U.N. (purple) for 14 randomly chosen space groups spanning the seven lattice types. **(b)** 14 randomly selected S.U.N. structures generated by MatterGen, one per space group in **(a)**.

D.7.1 Additional experimental details

For generating structures belonging to a target space group, we fine-tune our base model on the whole training set, and represent the latent embedding of the space group of a crystal via one-hot encoding of the space group.

We assess the capability of our model to correctly generate structures belonging to any space group via two tasks. For the first task (Fig. D8), we sample two space groups for each of the seven lattice systems, and choose to sample only from space groups that contain at least 1000 structures in the training set. We then compute the fraction of S.U.N. structures our fine-tuned model generates when conditioned on these space groups that are classified as belonging to that space group according to the `SpaceGroupAnalyzer` module of `pymatgen` [15, 34]. This metric is computed for 256 generated structures per space group after DFT relaxation has been performed. For the second task (Fig. D9), we generate 10,000 structures conditioned on space groups sampled randomly from the data distribution of the training set, and check whether

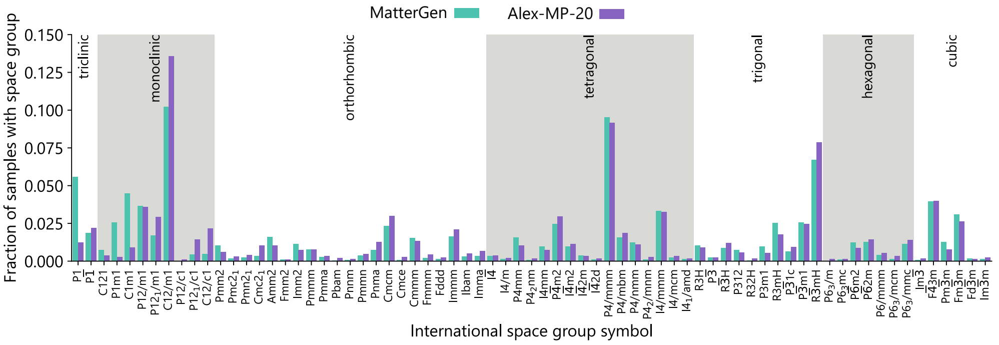

Fig. D9: **Generating materials with target symmetry.** Fraction of generated structures belonging to space groups generated by MatterGen fine-tuned on symmetry groups, sampled from the joint distribution of atoms in unit cell and space group of the training data (teal), and structures present in the training dataset (purple).

our model is able to reproduce the distribution of space groups from the training data. For both of the above tasks, the number of atoms in the systems are sampled from the distribution of number of atoms for that space group in the training set. This way, we avoid 'impossible' space group constraints, where the space group we condition on cannot be satisfied given the number of atoms we set.

D.7.2 Additional analysis of structures

The highlighted examples in Fig. D8 show a high degree of novelty, with few matching known prototypes. The combination of elements in generated structures is also not common, e.g., RbCuAgF, SrCaSiPt. It is therefore difficult to reference to known structures in literature. The majority of the examples could be assigned formal valences, and half were robust for their target symmetry even when symmetry was calculated with a higher symmetry-finding tolerance.

D.8 Designing materials with target magnetic, electronic and mechanical properties

This section provides supplementary information to the results in Section 2.4.

D.8.1 Additional experimental details

To generate structures conditioned on a target property, we fine-tune our base model on magnetic density ($N \approx$ 605,000 DFT labels), band gap ($N \approx$ 42,000) and bulk modulus ($N \approx$ 5,000), respectively. See Supplementary B.1 for more details on the fine-tuning scheme, and Supplementary D.1.2 for hyperparameter settings. We represent the latent embedding of scalar properties via a sinusoidal encoding from the Transformer architecture [35].

For each property in Fig. 4(a-c), we generate 512 samples with our fine-tuned model by conditioning on a value of 0.2 Å^{-3} for magnetic density, 3.0 eV for band gap, and

400 GPa for bulk modulus. We relax those structures using the MatterSim [45] MLFF and filter the relaxed structures by stability, uniqueness, and novelty. We then relax the remaining structures with DFT and filter by stability, uniqueness, and novelty. Finally, we compute the desired property of the remaining structures using DFT, and filter out structures where we consider the computed property to be an outlier, i.e., high bulk modulus values with more than 600 GPa and magnetic density values with more than 0.3 $Å^{-3}$. For more details on the DFT calculations, see Supplementary C.2. After all filters and DFT computations, we obtain 59 S.U.N. structures for magnetic density, 130 for band gap, and 19 for bulk modulus.

For Fig. 4(g), we use MatterGen to generate 15,360 samples conditioned on a magnetic density value of 0.2 $Å^{-3}$, relax those structures using MatterSim and filter the relaxed structures by stability, uniqueness, and novelty. This results in 163 candidate structures. Next, we relax them via DFT, with 140 of them being DFT stable. We then randomly sub-sample k structures according to the given DFT property calculation budget, and compute their magnetic density via DFT. As a baseline, we count the number of structures in the labeled training dataset that satisfy the target property constraint. For Fig. 4(h), we train a separate property predictor for bulk modulus (see details below), which we use for both MatterGen and the screening baseline. In particular, we use predicted bulk modulus values to fine-tune the base MatterGen model. We generate 8,192 samples conditioned on a bulk modulus value of 400 GPa, relax those structures using MatterSim and filter the relaxed structures by stability, uniqueness, and novelty. This results in 174 candidate structures. We relax those via DFT, with 131 of them being DFT stable. We then randomly sub-sample k structures according to the given DFT property calculation budget, and compute their bulk modulus via DFT. For the screening baseline, we use the bulk modulus property predictor to predict the bulk modulus values of all structures in the training dataset for which we do not have an existing DFT label, rank those structures by their predicted bulk modulus values, and choose the top k structures according the DFT property calculation budget. We then verify their actual bulk modulus values via DFT.

Property predictor details

The bulk modulus property predictor used in Fig. 4(h) consists of a GemNet-dT [6] encoder that provides atom and edge embeddings, followed by a mean readout layer. We employ three message-passing layers, a cutoff radius of 10 Å for the neighbor list construction, and set the dimension of nodes and edges hidden representations to 128.

To train the property predictor, we use all materials with DFT Voigt-Reuss-Hill average bulk modulus values from MP [14] (including structures with more than 20 atoms), which are 7,108 structures in total. We allocate 80% of the data for the training set, 10% for validation, and 10% for testing. We follow the MatBench benchmark [36] and predict the $\log_{10}$ bulk modulus. At the end of training, the model achieves a mean absolute error (MAE) of 9.5 GPa. The model was trained using the Adam optimizer. Gradient clipping was applied by value at 0.5. The learning rate was initialized at 5×10^{-4} and decayed using the ReduceLROnPlateau scheduler with decay factor 0.8, patience 10 and minimum learning rate 10^{-8}. The training was stopped when the validation loss stopped improving for 150 epochs.

D.8.2 Additional qualitative analysis of structures

Magnetic density conditional generation

For the high magnetic density generation task, a manual review of ten generated crystals chosen at random was performed, as well as the two representative structures with high magnetization density that were highlighted in Fig. 4. Of the random selection, eight of the ten generated structures were ordered approximations of a Fe-Co alloy: α-Fe with a partial Co substitution from 10% Co to 40% Co. The Fe-Co system is a well-known and versatile soft magnetic material system with a wide region where a α-$Fe_xCo_1{-}x$ phase is stable, with a mixture of γ-Fe and α-Co expected at lower Co content. While these generated structures could be considered "good", in the sense that they are physically plausible and indeed would be useful magnetic materials, they are likely only considered novel since these specific ordered unit cells do not currently exist in the reference databases. Also, as an alloy, predicted quantities such as energy above hull based on a single ordered approximation will be misleading. Two of the ten generated structures contained hydrogen, either as Fe_9H_2 or Fe_4Co_2H, with hydrogen in an ideal octahedral environment. The generation of these structures can be rationalized owing to extensive study of iron hydrides, with the generated structures having local atomic environments similar to that in a double hexagonal close-packed iron hydride structure that can exist at high pressure. The realization of these specific structures is unlikely with phase segregation expected to occur. The two structures highlighted in Fig. 4(d) contain Gd, an element with a large magnetic moment due to its 7 unpaired electrons when in its Gd^{3+} oxidation state and in its elemental state. Therefore, it is unsurprising that the model would preferentially generate materials containing Gd in this instance: as in the randomly selected sample, this task necessarily shows a strong compositional bias in the materials generated. The Gd_2N example is a layered material. Although the Gd_2N molecule has been studied [37], it is unknown if and how it might crystallize. The generated $Gd_6H_2CN_3$ structure is rocksalt-derived, with Gd on one site, and a mix of C, H and N on another site, with all atoms in almost ideal octahedral environments.

Band gap conditional generation

For the target band gap generation task, a manual review of ten generated crystals chosen at random was performed, as well as the two representative structures with desired target band gap that were highlighted in Fig. 4. Unlike the other single property optimization tasks, the generated crystals did not show a strong compositional dependence, with a wide range of elements presented in generated materials. All generated materials also could nominally charge balance, unlike the materials analyzed from other single property optimization tasks. This seems reasonable for a task designed to generate insulating systems; the target band gap of 3 eV (calculated with the PBE functional, and thus underestimating the true electronic band gap) should generate insulating materials, and thus more ionic solids. Of the random sample, we could only find $NaNO_3$ and the molecular crystal BI_3 as a compositions that had previously been synthesized. Both were in incorrect symmetry compared to their experimentally known structures, with the generated $NaNO_3$ in C2/c compared to the experimental $R\bar{3}c$, and BI_3 in P1 compared to the experimental $P6_3/m$. However, both had correct local

bonding and similar calculated band gaps to the band gaps calculated for their experimental structures. This indicates one instance whereby the model might still generate useful results even if the generated crystal structure is different from the experimental ground state, if it can guide a scientist towards investigation of a specific system.

The examples highlighted in Fig. 4(e) were $BiVO_4$ and $TlNO_3$. Of these, the local bonding in $BiVO_4$ was very similar to that in the experimentally-known bismuth vanadate which crystallizes in the $I4_1/amd$ [38] space group or $I4_1/a$ space group [39], unlike the generated Cmcm space group. The structure consists of ideal VO_4 tetrahedra and Bi co-ordinated by O atoms with all Bi-O bonds of similar length. However, the Bi atoms are displaced towards the face of its coordination polyhedra. While this new $BiVO_4$ polymorph is predicted to be stable by DFT, it would be surprising if it were synthesizable since $BiVO_4$ has already been extensively studied. It is possible the structure is dynamically unstable, kinetically inaccessible during growth, or there is simply a limitation in accuracy or precision of the DFT method used. However, since the DFT methods used here are predicting it to be stable and on the convex hull, it should not be seen as a failure of the model that this structure was generated. Likewise, $TlNO_3$ thallous nitrate is experimentally-known in the Pnma space group [40] with a calculated (PBE) band gap of 2.8 eV [41], however the generated crystal appears quite different to the experimental structure due to a change in Tl co-ordination around NO_3^- anions.

Bulk modulus conditional generation

For the high bulk modulus generation task, a manual review of ten generated crystals chosen at random was performed, as well as the two representative structures with high bulk modulus that were highlighted in Fig. 4(f). All structures show a strong compositional bias, containing a mix of refactory elements Re, W, Mo and Ir and frequently also B and C; this is consistent with literature on superhard materials (which includes materials with high bulk modulus). When the composition contains only refactory elements, the generated structure typically seems like an ordered approximation of an alloy of that composition, while those that also contain B or C typically take a very anisotropic, layered structure. As an example, Re_3B highlighted in Fig. 4 is known to exist in the Cmcm space group as an intermetallic and superconductor [42]. The Re_3B_2C example is more unusual, with layers of all Re, B, Re, C, Re, ..., and is a structure that nominally can charge balance. We note that the bulk modulus calculated is an averaged quantity over the full elastic tensor and we have not examined the directional bulk moduli in these highly anisotropic systems.

D.9 Designing low-supply-chain risk magnets

This section provides supplementary information to the results in Section 2.5.

D.9.1 Additional experimental details

To generate structures conditioned on magnetic density and HHI score, we fine-tune our base model on these two properties, encoded as in Supplementary D.8. To evaluate the performance of our model, we proceed as detailed in Supplementary D.8, and

generate 512 samples with our fine-tuned model, by conditioning on a magnetic density value of 0.2 $Å^{-3}$ and an HHI score of 1200. Of those, 85 samples remain after filtering by stability and uniqueness following the DFT relaxation. Finally, a total of 49 structures pass the novelty check w.r.t. the reference dataset and are reported in Fig. 5(a).

D.9.2 Additional qualitative analysis of structures

Targeting a low HHI index in addition to high magnetic density steers MatterGen away from generating structures with Co, which is associated with poor HHI scores. Example structures include $Fe_x Mn_{1-x} O$ rocksalt alloys ($MnFe_3O_4$, $Mn_2Fe_3O_5$) or additionally including Mg; however, they only exhibit a high magnetization density in a hypothetical ferromagnetic state, and not in their actual antiferromagnetic ground state. Other similar example outputs include a body-centered-cubic Fe_8Au system, which is well-known experimentally. While the joint optimization task could further be extended to produce more reasonable candidates—e.g., by penalizing expensive elements, and preferring metallic systems more likely to be ferromagnetic—the overall performance of the model with respect to the labels used for training is reasonable. It is possible that a better treatment of alloy systems would be required to improve performance of the high magnetic density generation task, and to ensure that the generated structures are truly novel.

D.9.3 Rediscovering experimental materials

Similar to the analysis in Supplementary D.5.4, we evaluate whether MatterGen can generate materials that are similar to experimentally known materials which it has not seen during training in order to assess if the model produces reasonable materials which could be synthesized. The reference dataset we employ comprises 6,126 ordered structures from the 'temporal' and 'i.i.d' ICSD splits mentioned in Supplementary D.5.4, and ~117.7k disordered ICSD structures (Fig. C3). We chose to examine conditional samples of the joint model in Section 2.5 that aims to generate samples of magnetic density 0.2 $Å^{-3}$ and an HHI score of 1250.

Of 130 samples whose corresponding DFT calculations completed successfully, 87 are not contained in the training set according to the *ordered-disordered* structure matcher. Of these structures, there are 26 that match at least one ICSD structure that is not contained in the training set. Of these 26 samples, 18 are unique according to the *ordered-disordered* structure matcher. There are 67 ICSD structures that match at least one of the aforementioned 18 samples, all of which are disordered materials. Of these 67 disordered ICSD structures, there are 12 sets in which all members are equivalent according to the *ordered-disordered* structure matcher (Table D8). We highlight matches to ICSD structures in chemical systems Fe-Ni, Co-Fe and Ca-Fe-Mn-O for which there are known permanent magnetic materials. Further, we note that all calculations of the total magnetic density performed in this work and of the training data were initialised in a ferromagnetic configuration. In general, it may be that other magnetic orderings, such as the antiferromagnetic ordering, are lower energy and are hence more likely to be synthesizable than the ferromagnetic one. This suggests that

a model for generating functional permanent magnets must be able to successfully predict the most stable magnetic ordering. Likewise, generated magnets would have to satisfy additional properties of interest such as a suitable critical temperature and high coercivity, which is related to its magneto-crystalline anisotropy. We acknowledge that these are complex properties that depend on many factors, which makes these properties difficult to simulate and predict. Solving these challenges would require substantial effort that is beyond the scope of this work.

MatterGen			ICSD			Experimental reference				
Formula(s)	Disordered Space Group(s)*	Magnetic Moment(s) (μ_B/atom)	Formula(s)	Space Group	Citation(s)	Formula(s)	Space Group(s)	Magnetic Moment(s) (μ_B/atom)	Magnetic Ordering	Citation
$Fe_{14}Co_1$<br>$Fe_{17}Co_1$	$Im\bar{3}m$<br>$Im\bar{3}m$	2.24<br>2.27	$Fe_{1.884}Co_{0.116}$	$Im\bar{3}m$	[43]	$Fe_{0.65}Co_{0.35}$	$Im\bar{3}m$	2.46	FM	[44]
$Fe_{10}Co_1$	$Im\bar{3}m$	2.28	$Fe_{2-x}Co_x$,<br>$x \in [0.18, 0.20]$	$Im\bar{3}m$	[45–47]	$Fe_{0.65}Co_{0.35}$	$Im\bar{3}m$	2.46	FM	[44]
$Fe_{15}Ni_1$	$Im\bar{3}m$	2.28	$Fe_{1.9}Ni_{0.1}$	$Im\bar{3}m$	[45, 48]	Fe<br>Fe_4Ni	$Im\bar{3}m$<br>$Pm\bar{3}m$	2.22<br>1.78	FM<br>FM	[44]<br>[44]
Fe_9Ni_1<br>$Fe_{11}Ni_1$	$Im\bar{3}m$<br>$Im\bar{3}m$	2.29<br>2.25	$Fe_{1.83}Ni_{0.17}$	$Im\bar{3}m$	[43]	Fe<br>Fe_4Ni	$Im\bar{3}m$<br>$Pm\bar{3}m$	2.22<br>1.78	FM<br>FM	[44]<br>[44]
Fe_9Ni_1	$Im\bar{3}m$	2.29	$Fe_{2-x}Ni_x$,<br>$x \in [0.17, 0.21]$	$P6_3/mmc$	[49, 50]	Fe<br>Fe_4Ni	$Im\bar{3}m$<br>$Pm\bar{3}m$	2.22<br>1.78	FM<br>FM	[44]<br>[44]
Fe_5O_6<br>Fe_9O_{10}<br>Fe_7O_8	P1<br>$P\bar{1}$<br>Pmmm	2.00<br>2.00<br>2.00	$Fe_{4-x-y}O_{4-y}$,<br>$x \in [0.10, 0.17]$,<br>$y \in \{0, 1\}$	$Fm\bar{3}m$	[51–57]					
Mn_5O_6	C2/m	2.09	$Mn_{4-x}O_4$,<br>$x \in [0.00, 0.36]$	$Fm\bar{3}m$	[58]					
$Mn_1Fe_8O_9$<br>$Mn_1Fe_9O_{10}$<br>$Mn_1Fe_7O_8$	C2/m<br>$P\bar{1}$<br>$Fm\bar{3}m$	2.06<br>2.06<br>2.05	$Mn_{0.404}Fe_{3.596}O_4$	$Fm\bar{3}m$	[59]					
$Mn_3Fe_2O_5$	C2/m	2.30	$Mn_{2.224}Fe_{1.748}O_4$	$Fm\bar{3}m$	[60]					
$Mn_5Fe_3O_8$	$Fm\bar{3}m$	2.31	$Mn_{2.676}Fe_{1.324}O_4$	$Fm\bar{3}m$	[59]					
$Mg_1Fe_4O_5$	I4/m<br>C2	1.60<br>1.60	$Mg_{0.956}Fe_{3.044}O_4$	$Fm\bar{3}m$	[59]					
$Ca_1Mn_4Fe_3O_{12}$	$R\bar{3}$	1.50	$Ca_2Mn_8Fe_6O_{24}$	$Im\bar{3}$	[61]	$Ca_2Mn_8Fe_6O_{24}$	$Im\bar{3}$	0.69	AFM	[61]

Table D8: Rediscovery of ICSD structures described in Supplementary D.9.3. Each row contains structures that are equivalent under the *ordered-disordered* structure matcher. Columns are grouped by the source of data, showing the formulas and space groups of members in each row, in addition to magnetic moment data if available. The rightmost column includes a non-exhaustive list of references of experimentally observed magnetic moments for materials that have similar formulas and space groups to those listed before.

* Space group of compositionally disordered equivalent structure, created by replacing elements that often form solid substitutions with their corresponding fractional occupancies.

D.10 Experimental validation of bulk modulus conditional generation

This section provides supplementary information to the results in Section 2.6.

D.10.1 Sampling details

We employ the same MatterGen model fine-tuned for bulk modulus conditional generation as detailed in Supplementary D.8.1, and generate 8192 samples for each of the following bulk modulus target values: 50, 100, 150, and 200 GPa, for a total of 32,768 samples. We furthermore add an elemental masking to disallow sampling of certain elements due to their toxicity, price, availability, or difficulty to experimentally handle; the excluded elements are: F, I, Cl, Br, C, N, Be, Sc, Rb, Rh, He, Pd, Cs, Pr, Nd, Pm, Tb, Dy, Ho, Tm, Yb, Os, Ir, Pt, Au, Re, Tl.

D.10.2 Rediscovery of ICSD structures

Acccording to our *ordered-disordered* structure matcher, we identify 101 structures among the 32,768 conditionally generated samples that match ordered ICSD structures in our Alex-MP-ICSD reference dataset. Note that none of these ICSD structures are included in our Alex-MP-20 training set. For all of them, we perform elastic tensor calculations using the DFT protocol highlighted in Supplementary C.2. We obtain 95 successful elastic tensor calculations and 6 non-converged ones.

D.10.3 Filtering steps to choose structures to synthesize

We apply the following filtering steps to the initial candidate set of 32,768 structures to narrow them down to four synthesis candidates.

1. We filter out compounds without oxygen to simplify the synthesis procedures, as oxides are often easier to synthesize. This reduces the number of candidates to 7919.
2. We filter out structures with a computed energy above hull above 0.05 eV/atom according to MatterSim [45]. This reduces the number of candidates to 2196.
3. We filter out duplicates and structures that match our Alex-MP-ICSD reference dataset, since we aim to synthesize a novel compound. This reduces the number of candidates to 551.
4. We filter out structures that display negative phonon modes when computing the phonon spectra using the MatterSim MLFF. The phonon spectra are computed using PhonoPy [62], with supercells contaning approximately 300 atoms, and using displacements of 0.03 Å. This reduces the number of candidates to 119.
5. We filter out structures with a computed energy above hull above 0.03 eV/atom according to DFT. This reduces the number of candidates to 75.
6. We rely on expert analysis for the final selection of candidates for synthesis. The criteria are: low number of elements, availability of precursors, predicted local ionic coordination. Four samples were chosen and synthesis was attempted for all of them.

Synthesis was successful for one of the four chosen samples, as detailed below.

D.10.4 $TaCr_2O_6$ experimental synthesis

The $TaCr_2O_6$ sample was synthesized via a conventional solid-state reaction where Cr powder ($\geq$ 99.99%, Sigma-Aldrich) and Ta2O5 ($\geq$ 99.99%, Ourchem) powder were used as starting materials, mixed in a molar ratio of 4:1 (Cr : Ta_2O_5). A high-energy planetary ball mill (Model: QM-3SP2) was employed to grind the mixture of Ta_2O_5 and Cr powders. The milling process utilized a 100 mL zirconia jar paired with zirconium oxide grinding beads, employing a mix of 3 mm and 5 mm beads in a ratio of 2:1. This mixed approach leverages the individual benefits of both larger and smaller beads, thereby optimizing grinding efficiency, particle size uniformity, and distribution. The bead-to-material ratio was maintained at 1:1.5, ensuring a sufficient amount of grinding media relative to the powder, while keeping approximately one-third of the jar's volume empty to facilitate proper milling dynamics. The mixture underwent high-energy milling at 500 rpm in alternating directions, with 1-hour intervals in each direction. This forward-reverse sequence was repeated for a total of 12 cycles, providing an effective method for uniform particle size reduction and ensuring thorough mixing of the material components. After ball milling, the sample was sintered in a muffle furnace (Model: KSL-1700X-A1) at 1200°C in air for 12 hours followed by pelletization at a pressure of 480 MPa. Finally, the pellets were sintered at 1500°C for 36 hours in the muffle furnace, yielding the final $TaCr_2O_6$ sample. The prepared $TaCr_2O_6$ sample was characterized using XRD on a Rigaku SmartLab SE system (see Supplementary D.10.6) and using x-ray photoelectron spectroscopy (XPS) on a Thermo Fisher K-Alpha X-ray photoelectron spectrometer system (see **??**).

D.10.5 Other attempted experimental syntheses

In addition to the $TaCr_2O_6$ sample, synthesis was attempted for three other compounds generated by MatterGen that passed all filtering steps detailed in Supplementary D.10.3, but was not successful. The compounds are Cr_2MoO_6 ($P4_2/mnm$), $LaMoO_4$ (C2/c), and Mn_3NiO_6 (R3), and they were generated conditionally with target bulk modulus values of 200, 100, and 150 GPa, respectively. They are shown in Fig. D10, and a brief description of their attempted synthesis is given below.

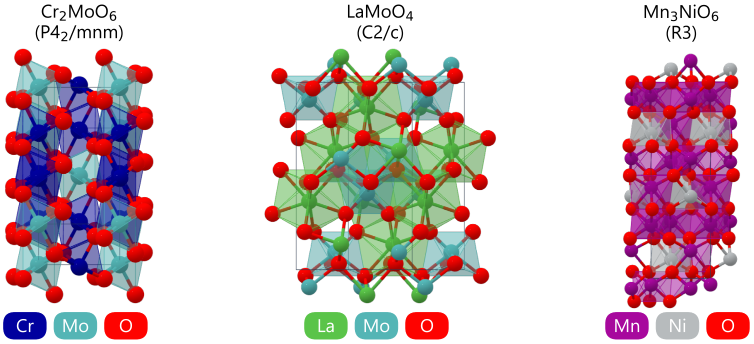

Fig. D10: **Other experimental synthesis candidates**

Cr_2MoO_6 ($P4_2/mnm$)

High-purity Cr_2O_3 powder ($\geq$ 99.99%, Sigma-Aldrich) and MoO_3 powder ($\geq$ 99.99%, Sigma-Aldrich) were used as the source materials, mixed in a molar ratio of 1:1 (Cr_2O_3 : MoO3). The mixture was processed using a high-energy planetary ball mill (model: QM-3SP2), following the procedure detailed in Supplementary D.10.4. After ball milling, the sample was sintered in a muffle furnace (Model: KSL-1700X-A1) at 750°C in air for 24 hours. The prepared powder samples were characterized using XRD on a Rigaku SmartLab SE system, but no peaks were observed. Furthermore, sintering at a higher temperature (900°C) has been tried, but a clear Mo loss was observed in this case. We note that Cr_2MoO_6 has been previously experimentally synthesized at high temperature and pressure in Collomb et al. [63]. The MatterGen-generated structure was identified as novel because the experimental entry is not listed in ICSD, and is therefore not included in our reference dataset.

$LaMoO_4$ ($C2/c$)

High-purity La_2O_3 powder (99.99 %, Sigma-Aldrich) and Mo powder (99.99 %, Sigma-Aldrich) were used as the source materials, mixed in a molar ratio of 1:2 (La_2O_3 : Mo). The mixture was processed using a high-energy planetary ball mill (model: QM-3SP2), following the procedure detailed in Supplementary D.10.4. After ball milling, the sample was air-annealed in a muffle furnace (model: KSL-1700X-A1) at 750°C for 24 hours. The prepared powder samples were characterized using XRD on a Rigaku SmartLab SE system, but no peaks were observed. Furthermore, sintering at a higher temperature (900°C) has been tried, but a clear Mo loss was observed in this case.

Mn_3NiO_6 ($R3$)

High-purity Mn_2O_3 powder ($\geq$ 99.99%, Sigma-Aldrich) and Ni_2O_3 powder ($\geq$ 99.99%, Sigma-Aldrich) were used as the source materials, mixed in a molar ratio of 3:1 (Mn_2O_3 : Ni_2O_3). The mixture was processed using a high-energy planetary ball mill (model: QM-3SP2), following the procedure detailed in Supplementary D.10.4. After ball milling, the sample was air-annealed in a muffle furnace (model: KSL-1700X-A1) at 1100°C for 24 hours. The prepared powder samples were characterized using XRD on a Rigaku SmartLab SE system. The XRD peaks did not match with the calculated ones for the predicted Mn_3NiO_6 structure. The observed peaks did instead strongly correlate with the ones of the known Mn_2NiO_4 phase [64].

D.10.6 Rietveld refinement

We carry out Rietveld refinement on the measured XRD spectra for the $TaCr_2O_6$ experimental sample using the FullProf program [65] (Fig. 6(a)). We obtain excellent agreement (chi-squared 3.596) with the measured XRD spectra by fitting the theoretical peaks of a mixture of two phases: a compositionally disordered $TaCr_2O_6$ Rutile phase (shown in Fig. 6(b)) and an ordered Cr_2O_3 Corundum phase. We obtain the structure for the $TaCr_2O_6$ disordered Rutile phase by replacing Cr and Ta atoms in the structure sampled by MatterGen with the corresponding partial occupancy sites (67% Cr and 33% Ta). From the Rietveld refinement we conclude that the $TaCr_2O_6$ disordered Rutile forms ~98% of the sample, while the ordered Cr_2O_3 Corundum phase

forms the remaining ∼2%. While conducting the Rietveld refinement, the following alternative structures on or near the Ta-Cr-O convex hull were tested for goodness of fit: Cr (Im$\bar{3}$m, ordered), CrO_2 (P4_2/mnm, ordered), $TaCr_2O_6$ (P4_2/mnm, ordered), Ta_2O_5 (Pmmm, ordered), $TaCrO_4$ (I4_1md, ordered), $TaCrO_4$ (I4_1md, disordered), Ta_2CrO_6 (P4_2/mnm, ordered), Ta_2CrO_6 (P4_2/mnm, disordered), but none of them matched the observed XRD spectra, even partially.

D.10.7 X-ray photoelectron spectroscopy

We perform an XPS analysis on the synthesized $TaCr_2O_6$ powder sample to verify the chemical composition and state of the elements (Fig. D11). The measurement was

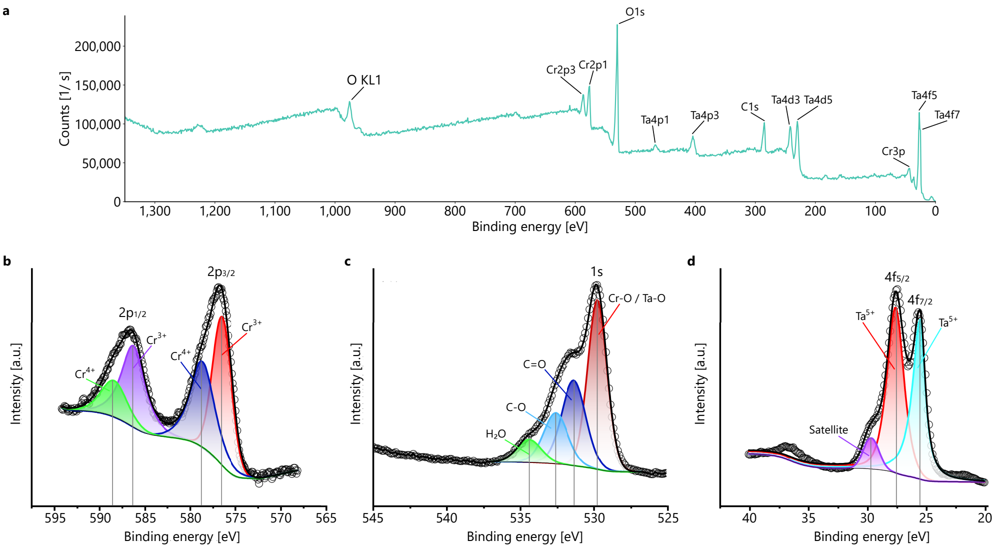

Fig. D11: **XPS measurements for the $TaCr_2O_6$ powder sample. (a)** Full XPS measurement for the sample, with annotation of major peaks. **(b-d)** XPS analysis for the Cr2p, O1s, and Ta4f peaks, respectively. The black circles indicate the measured electron count, the black lines indicate the fitted electron count, and the colored Gaussians highlight the single peaks. Source plots are obtained from the Thermo Fisher Avantage software. Vertical gray lines act as a visual aid to highlight the binding energy of each fitted peak.

taken four months after sample preparation and XRD analysis, using a Thermo Fisher K-Alpha X-ray Photoelectron Spectrometer System, with a spot size of 400 μm and a monochromatic Al K-Alpha source. We used an energy step size of 1 eV, a pass energy of 100.0 eV, and 1361 energy steps for the full spectrum sweep displayed in Fig. D11(a). For the three focused scans we instead employed an energy step size of 0.1 eV, a pass energy of 50.0 eV and a variable number of steps: 261 for the Cr2p sweep (Fig. D11(b)) and 201 for both the O1s (Fig. D11(c)) and Ta4f sweeps (Fig. D11(d)).

The total acquisition times were 68 s for the full sweep, 104 s for the Cr2p sweep, 60 s for the O1s sweep, and 80 s for the Ta4f sweep. The peak fitting analysis was carried out on the Thermo Fisher Avantage software.

Our analysis indicates the presence of Cr in both 4+ and 3+ valence states (Fig. D11(b)), and of Ta in a 5+ valence state (Fig. D11(d)), hence corroborating the hypothesis that the structure we synthesize has reduced formula $TaCr_2O_6$. The Cr2p core level (Fig. D11(b)) is fitted with two spin orbit doublets, one for Cr^{3+} and one for Cr^{4+}. We measure the two peaks belonging to $Cr^{3+}2p_{3/2}$ and $Cr^{3+}2p_{1/2}$ at binding energy values of 576.6 eV and 586.4 eV, respectively, and the two peaks belonging to $Cr^{4+}2p_{3/2}$ and $Cr^{4+}2p_{1/2}$ at binding energy values of 578.8 eV and 588.6 eV, respectively. The O1s core level in Fig. D11(c) is fitted with four peaks, which we identify as Cr-O / Ta-O (529.8 eV), C=O (531.4 eV), C−O (532.6 eV), and H_2O (534.4 eV). These last three peaks could indicate the presence of adsorbed CO_2 and H_2O in the surface layers of the sample. The Ta4f core level in Fig. D11(d) is fitted with one spin orbit doublet. We measure the two peaks belonging to $Ta^{5+}4f_{7/2}$ and $Ta^{5+}4f_{5/2}$ at binding energy values of 25.5 eV and 27.7 eV, respectively. We additionally measure a peak at 29.7 eV, which we hypothesize to be a satellite peak, and a possible peak around 37.0 eV, which could belong to $Ta^{5+}5p_{3/2}$.

D.10.8 Young's modulus measurements via nanoindentation

We experimentally measure the Young's modulus value of the prepared $TaCr_2O_6$ sample via nanoindentation. The nanoindentation tests are carried out using a iMacro nanoindentation tester equipped with a diamond Berkovich tip, and using a data acquisition frequency of 100 Hz. The maximum load force is 1 mN. In Fig. D12 we report the load-force plots for four measurements carried out in different locations of the synthesized $TaCr_2O_6$ pellet. From the measured unloading slopes we obtain the

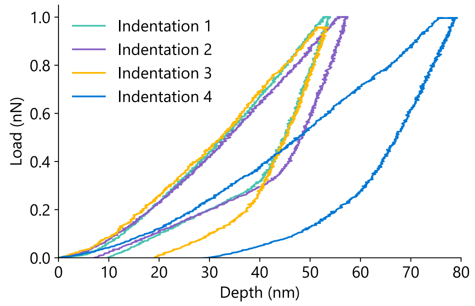

Fig. D12: Load/depth profile for the nano indentation measurements on four locations of the $TaCr_2O_6$ pellet we experimentally synthesize.

following four Young's modulus measurements: 184.7, 200.6, 202.4, and 169.3 GPa. To compute the above, we assumed a Poisson ratio value for the sample of 0.30, which we obtain from the DFT elastic modulus calculation we performed. We can then compute

the bulk modulus K values for the sample as:

$$K = \frac{Y}{3(1-2\sigma)}, \tag{D50}$$

where Y is the Young's modulus and σ the Poisson ratio. From Eq. (D50) we obtain the following bulk modulus values for the sample: 153.9, 167.2, 168.7, and 141.1 GPa, which result in a maximum of 168.7 GPa and an average of 157.7±11.2 GPa. Since correct measurement of Young's modulus requires a compact sample, we hypothesize that the observed variation in measurements stems from local non-compactness of the powder sample we synthesize. Therefore, we take the maximum measured Young's modulus as the best estimate of the true Young's modulus for this sample. The corresponding bulk modulus value (168.7 GPa) is close to the target bulk modulus value we condition on to generate this compound (200 GPa), with a 16% relative difference.

Supplementary References

[1] Sohl-Dickstein, J., Weiss, E., Maheswaranathan, N., Ganguli, S.: Deep unsupervised learning using nonequilibrium thermodynamics. In: International Conference on Machine Learning, pp. 2256–2265 (2015). PMLR

[2] Austin, J., Johnson, D.D., Ho, J., Tarlow, D., Berg, R.: Structured denoising diffusion models in discrete state-spaces. Advances in Neural Information Processing Systems **34**, 17981–17993 (2021)

[3] Song, Y., Ermon, S.: Improved techniques for training score-based generative models. Advances in Neural Information Processing Systems **33**, 12438–12448 (2020)

[4] Jing, B., Corso, G., Chang, J., Barzilay, R., Jaakkola, T.: Torsional diffusion for molecular conformer generation. Advances in Neural Information Processing Systems **35**, 24240–24253 (2022)

[5] Hoogeboom, E., Satorras, V.G., Vignac, C., Welling, M.: Equivariant diffusion for molecule generation in 3D. In: International Conference on Machine Learning, pp. 8867–8887 (2022). PMLR

[6] Gasteiger, J., Becker, F., Günnemann, S.: Gemnet: Universal directional graph neural networks for molecules. Advances in Neural Information Processing Systems **34**, 6790–6802 (2021)

[7] Niggli, P., Wien, W.: Kristallographische und Strukturtheoretische Grundbegriffe - 7/1. Handbuch der Experimentalphysik, pp. 108–176. Akademische Verlagsgesellschaft, Leipzig (1928)

[8] Kingma, D.P., Welling, M.: Auto-Encoding Variational Bayes. In: Bengio, Y., LeCun, Y. (eds.) 2nd International Conference on Learning Representations, ICLR 2014, Banff, AB, Canada, April 14-16, 2014, Conference Track Proceedings (2014). http://arxiv.org/abs/1312.6114

[9] Rombach, R., Blattmann, A., Lorenz, D., Esser, P., Ommer, B.: High-resolution image synthesis with latent diffusion models. In: Proceedings of the IEEE/CVF Conference on Computer Vision and Pattern Recognition, pp. 10684–10695 (2022)

[10] Satorras, V.G., Hoogeboom, E., Welling, M.: E(n) equivariant graph neural networks. In: International Conference on Machine Learning, pp. 9323–9332 (2021). PMLR

[11] Liu, N., Li, S., Du, Y., Torralba, A., Tenenbaum, J.B.: Compositional visual generation with composable diffusion models. In: European Conference on Computer Vision, pp. 423–439 (2022). Springer

[12] Materials Project: GitHub - materialsproject/emmet. https://github.com/materialsproject/emmet

[13] Wang, A., Kingsbury, R., McDermott, M., Horton, M., Jain, A., Ong, S.P., Dwaraknath, S., Persson, K.A.: A framework for quantifying uncertainty in DFT energy corrections. Scientific reports **11**(1), 15496 (2021)

[14] Kresse, G., Furthmüller, J.: Efficient iterative schemes for ab initio total-energy calculations using a plane-wave basis set. Physical review B **54**(16), 11169 (1996)

[15] Ong, S.P., Richards, W.D., Jain, A., Hautier, G., Kocher, M., Cholia, S., Gunter, D., Chevrier, V.L., Persson, K.A., Ceder, G.: Python materials genomics (pymatgen): A robust, open-source python library for materials analysis. Computational Materials Science **68**, 314–319 (2013)

[16] Perdew, J.P., Burke, K., Ernzerhof, M.: Generalized gradient approximation made simple. Physical review letters **77**(18), 3865 (1996)

[17] Riebesell, J.: Pymatviz: visualization toolkit for materials informatics. 10.5281/zenodo.7486816 - https://github.com/janosh/pymatviz (2022). https://doi.org/10.5281/zenodo.7486816 . https://github.com/janosh/pymatviz

[18] Kingma, D.P., Ba, J.: Adam: A method for stochastic optimization. arXiv preprint arXiv:1412.6980 (2014)

[19] Bartel, C.J.: Review of computational approaches to predict the thermodynamic stability of inorganic solids. Journal of Materials Science **57**(23), 10475–10498 (2022)

[20] Aykol, M., Dwaraknath, S.S., Sun, W., Persson, K.A.: Thermodynamic limit for synthesis of metastable inorganic materials. Science advances **4**(4), 0148 (2018)

[21] Isaacs, E.B., Wolverton, C.: Performance of the strongly constrained and appropriately normed density functional for solid-state materials. Physical Review Materials **2**(6), 063801 (2018)

[22] Marsh, R.E.: P1 or P$\bar{1}$? Or something else? Acta Crystallographica Section B: Structural Science **55**(6), 931–936 (1999)

[23] Antoniuk, E.R., Cheon, G., Wang, G., Bernstein, D., Cai, W., Reed, E.J.: Predicting the synthesizability of crystalline inorganic materials from the data of known material compositions. npj Computational Materials **9**(1), 155 (2023)

[24] Himanen, L., Rinke, P., Foster, A.S.: Materials structure genealogy and high-throughput topological classification of surfaces and 2D materials. npj Computational Materials **4**(1), 52 (2018)

[25] Horton, M., Shen, J.-X., Burns, J., Cohen, O., Chabbey, F., Ganose, A.M., Guha,

R., Huck, P., Li, H.H., McDermott, M., Montoya, J., Moore, G., Munro, J., O'Donnell, C., Ophus, C., Petretto, G., Riebesell, J., Wetizner, S., Wander, B., Winston, D., Yang, R., Zeltmann, S., Jain, A., Persson, K.A.: Crystal Toolkit: A Web App Framework to Improve Usability and Accessibility of Materials Science Research Algorithms (2023)

[26] Pan, H., Ganose, A.M., Horton, M., Aykol, M., Persson, K.A., Zimmermann, N.E., Jain, A.: Benchmarking coordination number prediction algorithms on inorganic crystal structures. Inorganic chemistry **60**(3), 1590–1603 (2021)

[27] Callister, W.D., Rethwisch, D.G., Blicblau, A., Bruggeman, K., Cortie, M., Long, J., Hart, J., Marceau, R., Mitchell, R.: Materials Science and Engineering: an Introduction vol. 7. John wiley & sons New York, ??? (2007)

[28] Ganose, A.M., Jain, A.: Robocrystallographer: automated crystal structure text descriptions and analysis. MRS Communications **9**(3), 874–881 (2019)

[29] Nekrasov, I.A., Keller, G., Kondakov, D., Kozhevnikov, A., Pruschke, T., Held, K., Vollhardt, D., Anisimov, V.: Comparative study of correlation effects in CaVO3 and SrVO3. Physical Review B **72**(15), 155106 (2005)

[30] Pati, B., Choudhary, R., Das, P.R.: Phase transition and electrical properties of strontium orthovanadate. Journal of alloys and compounds **579**, 218–226 (2013)

[31] Zavalij, P.Y., Whittingham, M.S.: Structural chemistry of vanadium oxides with open frameworks. Acta Crystallographica Section B: Structural Science **55**(5), 627–663 (1999)

[32] Tang, F., Po, H.C., Vishwanath, A., Wan, X.: Comprehensive search for topological materials using symmetry indicators. Nature **566**(7745), 486–489 (2019)

[33] Smidt, T.E., Mack, S.A., Reyes-Lillo, S.E., Jain, A., Neaton, J.B.: An automatically curated first-principles database of ferroelectrics. Scientific data **7**(1), 72 (2020)

[34] Grosse-Kunstleve, R., Adams, P.: Algorithms for deriving crystallographic space-group information. II. Treatment of special positions. Acta crystallographica. Section A, Foundations of crystallography **58**, 60–5 (2002) https://doi.org/10.1107/S0108767301016658

[35] Vaswani, A., Shazeer, N., Parmar, N., Uszkoreit, J., Jones, L., Gomez, A.N., Kaiser, Ł., Polosukhin, I.: Attention is all you need. Advances in Neural Information Processing Systems **30** (2017)

[36] Dunn, A., Wang, Q., Ganose, A., Dopp, D., Jain, A.: Benchmarking materials property prediction methods: the Matbench test set and Automatminer reference

algorithm. npj Computational Materials **6**(1), 138 (2020)

[37] Willson, S.P., Andrews, L.: Characterization of the reaction products of laser-ablated early lanthanide metal atoms with dinitrogen. infrared spectra of LnN, LnN2, (LnN) 2, and Ln(NN)x molecules. The Journal of Physical Chemistry A **102**(50), 10238–10249 (1998)

[38] Dreyer, E. G.; Tillmanns: Dreyerit: Ein natürliches, tetragonales Wismutvandat von Hirschhorn/Pfalz. Neues Jahrb. Mineral., Monatsh. (4), 151–154 (1981)

[39] Sleight, A., Chen, H.-Y., Ferretti, A., Cox, D.: Crystal growth and structure of BiVO4. Materials Research Bulletin **14**(12), 1571–1581 (1979)

[40] Fraser, W., Kennedy, S., Snow, M.: The nitrate positions in phase III of thallous nitrate. Acta Crystallographica Section B: Structural Crystallography and Crystal Chemistry **31**(2), 365–370 (1975)

[41] Materials Project: mp-5915: TlNO3 (Orthorhombic, Pnma, 62). https://doi.org/10.17188/1277176 (2023). https://doi.org/10.17188/1277176

[42] Kawano, A., Mizuta, Y., Takagiwa, H., Muranaka, T., Akimitsu, J.: The Superconductivity in Re–B System. Journal of the Physical Society of Japan **72**(7), 1724–1728 (2003) https://doi.org/10.1143/jpsj.72.1724

[43] Zwell, L.: Effects of Co, Cr, Ir, Pt, Re, Rh, and Ru on the lattice parameter and density of alpha iron. Metallurgical and Materials Transactions B: Process Metallurgy and Materials Processing Science **4**, 1990–1992 (1973)

[44] Coey, J.M.D.: Magnetism and Magnetic Materials. Cambridge University Press, ??? (2010)

[45] Yamamoto, A.: Modulated structure of wustite (Fe1-X O) (Threedimensional modulation). Acta Crystallographica, Section B: Structural Crystallography and Crystal Chemistry **38**, 1451–1456 (1982)

[46] Jay, A.H.: Note on oxide systems pertaining to steel-making furnace slags. FeO-MnO, FeO-MgO, CaO-MnO, MgO-MnO. Journal of the Iron and Steel Institute, London **152**, 15–18 (1945)

[47] Guo, J.: High-pressure synthesis of A-site ordered perovskite CaMn_3(fe_3mn)o_12 and sequential long-range antiferromagnetic ordering and spin glass transition. Journal of Solid State Chemistry **278**, 1–5 (2019)

[48] Gavarri, J.R.: Cristallochimie - evolution structurale de la wuestite solide: Variation du facteur d'agitation thermique isotrope moyen avec la temperature et la composition d'equilibre. Comptes Rendus des Seances de l'Academie des Sciences, Serie C: Sciences Chimiques **284**, 3353–3373 (1977)

[49] Hope, D.A.O.: A neutron diffraction, magnetic susceptibility, and Mossbauer-effect study of (Mnx Fe1-x)y o solid solutions. Inorganic Chemistry **21**, 2804–2809 (1982)

[50] Willis, B.T.M.: Change of structure of ferrous oxide at low temperature. Acta Crystallographica **6**, 827–831 (1953)

[51] Radler, M.J.: The defect structures of Mn1-xO. Journal of Physics and Chemistry of Solids **53**, 141–154 (1992)

[52] Sutton, A.L.: The lattice spacings of solid solutions of titanium, vanadium, chromium, manganese, cobalt, and nickel in alpha-iron. Philosophical Magazine **46**, 1295–1309 (1955)

[53] Gulyaev, A.P.: Mechanisms of property variations in solid solutions. Zhurnal Tekhnicheskoi Fiziki **20**, 66–78 (1950)

[54] Takahashi, T.: Isothermal compression of the alloys of iron up to 300 kbar at room temperature: Iron-nickel alloys. Journal of Geophysical Research, B **73**, 4717–4725 (1968)

[55] Jette, E.R.: An x-ray study of the wuestite (Fe O) solid solutions. Journal of Chemical Physics **1**, 29–36 (1933)

[56] Akimitsu, M.: Magnetic structure and magnetic properties of non-stoichiometric Fe1-xO. Journal of Physics and Chemistry of Solids **44**, 497–505 (1983)

[57] Radler, M.J.: Point defect clusters in wuestite. Journal of Physics and Chemistry of Solids **51**, 217–228 (1990)

[58] Mukhamedov, B.O.: Spinodal decomposition in ternary Fe-Cr-Co system. Journal of Alloys and Compounds **695**, 250–256 (2017)

[59] Gorton, A.T.: Thermal expansion coefficients for iron and its oxides from X-ray diffraction measurements at elevated temperatures. Transactions of the Metallurgical Society of AIME **233**, 1519–1525 (1965)

[60] Komabayashi, T.: In situ x-ray diffraction measurements of the fcc-hcp phase transition boundary of an Fe-Ni alloy in an internally heated diamond anvil cell. Physics and Chemistry of Minerals **39**, 329–338 (2012)

[61] Abrahamson, E.P.: The lattice parameters and solubility limits of alpha iron as affected by some binary transition-element additions. Transactions of the Metallurgical Society of AIME **236**, 76–87 (1966)

[62] Togo, A., Chaput, L., Tadano, T., Tanaka, I.: Implementation strategies in phonopy and phono3py. J. Phys. Condens. Matter **35**(35), 353001 (2023) https://doi.org/10.1088/1361-648X/acd831

[63] Collomb, A., Capponi, J., Gondrand, M., Joubert, J.: Synthese de quelques oxydes mixtes de type A6+ B23+ O6 en milieu hydrothermal sous tres haute pression. Journal of Solid State Chemistry **23**(3-4), 315–319 (1978)

[64] Medany, S.S., Hefnawy, M.A., Kamal, S.M.: High-performance spinel nimn2o4 supported carbon felt for effective electrochemical conversion of ethylene glycol and hydrogen evolution applications. Scientific Reports **14**(1), 471 (2024)

[65] Rodriguez-Cavajal, J.: Recent developments of the program FULLPROF. Comm. Powder Diffract. Newsl. **26**, 12 (2001)
